# Disrupted resolution mechanisms favour altered phagocyte responses in COVID-19

**DOI:** 10.1161/CIRCRESAHA.121.319142

**Published:** 2021-07-09

**Authors:** Duco Steven Koenis, Issa Beegun, Charlotte Camille Jouvene, Gabriel Amador Aguirre, Patricia Regina Souza, Maria Gonzalez-Nunez, Lucy Ly, Kimberly Pistorius, Hemant M. Kocher, William Ricketts, Gavin Thomas, Mauro Perretti, Ghassan Alusi, Paul Pfeffer, Jesmond Dalli

**Affiliations:** 1 William Harvey Research Institute, Barts and The London School of Medicine and Dentistry, Queen Mary University of London, UK; 2 Barts Cancer Institute, Barts and The London School of Medicine and Dentistry, Queen Mary University of London, UK; 3 Department of Respiratory Medicine, Barts Health NHS Trust, London, UK; 4 Centre for Inflammation and Therapeutic Innovation, Queen Mary University of London, UK

**Keywords:** basic science research, inflammation, cellular reprogramming, COVID-19, leukocytes, lipid mediators, inflammation

## Abstract

**Rationale:**

Resolution mechanisms are central in both the maintenance of homeostasis and the return to catabasis following tissue injury and/or infections. Amongst the pro-resolving mediators, the essential fatty acid-derived specialized pro-resolving lipid mediators (SPM) govern immune responses to limit disease severity. Notably, little is known about the relationship between the expression and activity of SPM pathways, circulating phagocyte function and disease severity in patients infected with novel severe acute respiratory syndrome coronavirus 2 (SARS-CoV-2) leading to coronavirus disease 2019 (COVID-19).

**Objective:**

Herein, we investigated the link between circulating SPM concentrations and phagocyte activation status and function in COVID-19 patients (n=39) compared to healthy (n=12) and post-COVID-19 (n=8) volunteers.

**Methods and Results:**

Lipid mediator profiling demonstrated that plasma SPM concentrations were upregulated in patients with mild COVID-19 and are downregulated in those with severe disease. SPM concentrations were correlated with both circulating phagocyte activation status and function. Perturbations in plasma SPM concentrations and phagocyte activation were retained after the resolution of COVID-19 clinical symptoms. Treatment of patients with dexamethasone upregulated both the expression of SPM biosynthetic enzymes in circulating phagocytes and plasma concentration of these mediators. Furthermore, incubation of phagocytes from COVID-19 patients with SPM rectified their phenotype and function. This included a downregulation in the expression of activation markers, a decrease in the Tissue Factor and inflammatory cytokine expression, and an upregulation of bacterial phagocytosis.

**Conclusions:**

The present findings suggest that downregulation of systemic SPM concentrations is linked with both increased disease severity and dysregulated phagocyte function. They also identify the upregulation of these mediators by dexamethasone as a potential mechanism in host protective activities elicited by this drug in COVID-19 patients. Taken together, our findings elucidate a role for altered resolution mechanisms in the disruption of phagocyte responses and the propagation of systemic inflammation in COVID-19.

## Introduction

The inflammatory response is an essential protective mechanism that evolved to safeguard the host from invading pathogens and facilitate the restoration of tissue function through repair and regeneration of damaged tissues^1–4^. This finely tuned response becomes unravelled in disease, often resulting in disseminated inflammation. One such example is coronavirus disease 2019 (COVID-19) caused by the novel severe acute respiratory syndrome coronavirus 2 (SARS-CoV-2)^5^. Current evidence suggests that in a large part of the population, SARS-CoV-2 infections result in either no or mild symptoms that do not require hospitalization. However, in the remainder of cases, SARS-CoV-2 results in moderate to severe disease requiring hospitalization, with a subset of these patients becoming critically ill and requiring ventilation support.

COVID-19 is characterized by a number of clinical presentations that in severe cases include pneumonia, acute respiratory distress syndrome and multiple organ failure^5^. This disseminated inflammatory response is thought to involve alveolar damage that can lead to cardiovascular complications and multi-organ failure. A significant body of effort has now gone into understanding the mechanisms underpinning this dysregulated systemic inflammatory response, since insights into these mechanisms may provide novel therapeutic leads for the treatment of hospitalized patients. This notion is underpinned by results obtained in the RECOVERY Trial, which demonstrated a significant reduction in mortality in critically ill patients treated with the anti-inflammatory corticosteroid dexamethasone^6^.

Initial efforts into detailing these inflammatory mechanisms found a marked upregulation of circulating pro-inflammatory cytokines as well as an increased inflammatory phenotype of innate immune cells, primarily monocytes, in COVID-19 patients with severe disease^7–12^. This phenotype is reminiscent of the dysregulated resolution responses observed following infections by other viral and bacterial pathogens^1–4^. These findings suggest that in addition to an increase in pro-inflammatory mediator production there may also be disruptions in resolution pathways in COVID-19 patients.

Resolution mechanisms are orchestrated by a number of endogenous autacoids that include the omega-3 fatty acid derived specialized pro-resolving lipid mediators (SPM)^4, 13^. These molecules are classified into four distinct families: lipoxins, resolvins, protectins and maresins, which are produced *via* the stereoselective conversion of essential fatty acids. SPM display both host-directed as well as anti-viral actions during viral infections. For example, the docosahexaenoic acid (DHA)-derived Resolvin (Rv)D1 and its precursor 17-hydroxy-docosahexaenoic acid regulate B-cell response during H1N1 infections in mice by promoting an antibody class switch^14^. The Protectin (PD) family of mediators regulate viral propagation by inhibiting intracellular viral RNA transport mechanisms^15^. Similarly, the eicosapentaenoic acid (EPA)-derived RvE1 reduces effector T-cell and neutrophil-mediated propagation of inflammation during herpes simplex virus infections^16^. Furthermore, recent studies demonstrate that circulating concentrations of these mediators are linked with disease outcome in sepsis and in treatment responsiveness in humans with chronic inflammatory conditions such as rheumatoid arthritis^17, 18^.

Given the immunoregulatory actions of SPM and the potential diagnostic and prognostic value of measuring peripheral levels of these molecules, we sought to evaluate whether SPM pathways are altered in COVID-19 patients. We also investigated the impact that disruptions in these pathways may have on phagocyte biology and disease propagation in COVID-19 patients.

## Methods

All data supporting the findings of this study are available from the corresponding author upon reasonable request. Please see the Supplemental Materials for the Expanded Materials and Methods and Major Resources Table.

## Results

### Altered peripheral blood LM profiles in COVID-19 pneumonia patients

**Figure 1:**
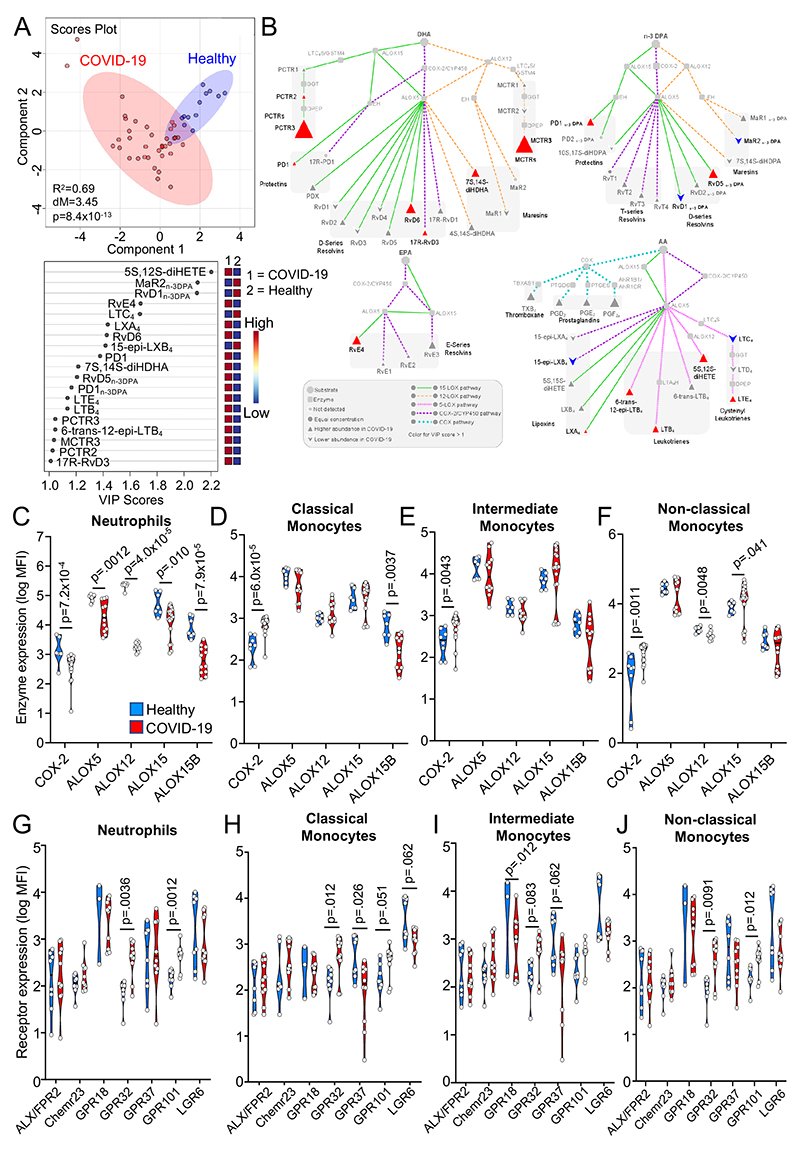
Upregulation of SPM pathways in COVID-19 patients. Peripheral blood was collected from COVID-19 patients or healthy volunteers and (**A-B**) plasma lipid mediators were identified and quantified using LC-MS/MS-based mediator profiling (n=38 COVID-19 patients, n=12 healthy volunteers). (**A**) PLS-DA was performed on identified mediators. *Top panel* – Scores plot, shaded area represents 95% confidence interval (CI), R^2^ = coefficient-of-determination, p = p-value from Hotelling’s T-squared test, dM = Mahalanobis distance between groups. *Bottom Panel* – VIP scores plot. (**B**) Analysis highlighting mediators with VIP scores >1 in PLS-DA and their biosynthetic pathways. (**C-J**) Whole blood from healthy volunteers and COVID-19 patients was incubated with lineage-marker antibodies against neutrophils (CD16+) and monocyte subsets (classical CD14+CD16+, intermediate CD14^++^CD16^++^, non-classical CD14^+^CD16^+^) in combination with antibodies against (**C-F**) lipid mediator biosynthetic enzymes or (**G-J**) SPM receptors, and their expression was evaluated using flow cytometry. For (**C-F**), n=19 COVID-19 patients (for COX-2, ALOX15), n=12 COVID-19 patients (for ALOX5, ALOX12, ALOX15B) and n=7 Healthy volunteers. For (**G-J**), n=11 COVID-19 patients (except for GPR18 and GPR37 where n=10) and n=8 Healthy volunteers (except for GPR18 where n=3 Healthy volunteers). Statistical differences between Healthy and COVID-19 groups were established using Mann-Whitney Test for each molecule and raw p-values are displayed.

In order to evaluate whether SPM concentrations are differentially regulated in COVID-19 patients we first assessed plasma lipid mediator (LM) profiles in hospitalized patients with COVID-19 (n=38) and compared these with LM profiles obtained from healthy volunteers with no clinical suggestion of COVID-19 infection (n=12; see Supplemental Table I for clinical characteristics). Using liquid chromatography tandem mass spectrometry (LC-MS/MS), we identified LM from all four bioactive metabolomes in both hospitalized patients and healthy volunteers. We then assessed their relative levels using Partial Least Squares Discriminant analysis (PLS-DA), a dimensionality-reducing multivariate analysis that creates a linear regression model accounting for multicollinearity and identifies the relationship between samples based on LM concentrations. This demonstrated that LM profiles in hospitalized COVID-19 patients were different from those found in plasma from healthy volunteers, as illustrated by a separate clustering of LM profiles between the two groups (Figure 1A and Supplemental Table II). Assessment of Variable Importance in Projection (VIP) scores, which identify those mediators that contribute most to the observed separation between the two groups, showed that 19 mediators displayed a VIP score >1 (Figure 1A). Intriguingly, the majority of mediators found to be differentially regulated between COVID-19 patients and healthy volunteers belonged to the SPM family. This included an upregulation of SPM from the DHA, n-3 docosapentaenoic acid (DPA), and EPA bioactive metabolomes in COVID-19 patients, such as DHA-derived MCTR3, PCTR3, and RvD6, and n-3 DPA-derived RvD5_n-3 DPA_ and PD1_n-3 DPA_.

To obtain insights into potential changes in LM biosynthetic pathways in COVID-19 patients, we next performed a pathway analysis on mediators that displayed the greatest differences in concentrations between the two groups (i.e. VIP scores >1). This demonstrated an upregulation of 5-Lipoxygenase (ALOX5)–ALOX15 interaction products as highlighted by increases in RvD6, RvD5_n-3__DPA_ and RvE4, and a decrease in cytochrome (CY)P450–ALOX5 interaction products evidenced by a downregulation in 15-epi-LXB_4_ in COVID-19 patients when compared with healthy volunteers (Figure 1B). Together these findings provide evidence for altered LM, in particular SPM, levels in COVID-19 patients.

### Differential regulation of SPM biosynthetic enzymes and receptors in peripheral blood phagocytes from COVID-19 patients

Phagocytes play a central role in LM production^19, 20^. Having identified changes in plasma concentrations of mediators from both ALOX and COX biosynthetic pathways, we next investigated whether these enzymes were differentially expressed in peripheral blood phagocytes from patients with COVID-19 when compared with those from healthy volunteers. Flow cytometric analysis of peripheral blood neutrophils from patients with COVID-19 demonstrated a significant downregulation in the expression of all LM biosynthetic enzymes analysed (Figure 1C, Supplemental Figure I-A). On the other hand, in peripheral blood monocytes we observed an overall trend towards increased expression of these enzymes, which reached statistical significance for COX-2 in all three monocyte subsets and for ALOX15 in non-classical monocytes (Figure 1D-F). Together these findings uncover a differential regulation in the expression of LM biosynthetic enzymes in circulating phagocytes from COVID-19 patients.

The biological actions of SPM are mediated by cognate G protein-coupled receptors (GPCR)^21,22^. Thus, we next evaluated whether phagocytes from COVID-19 patients displayed altered expression of SPM receptors. Flow cytometric assessment of SPM receptors in peripheral blood neutrophils demonstrated a significant upregulation of GPR32 and GPR101 (Figure 1G). The former receptor mediates the protective actions of RvD1, 17R-RvD1, RvD3, 17R-RvD3, RvD5, 15-epi-LXA4 and LXA_4_^21, 22^, whereas the latter is the cognate receptor for RvD5_n-3 DPA_^23^. GPR32 was upregulated in all three monocytes subsets, while GPR101 was upregulated in classical and non-classical monocytes (Figure 1H-J). We also observed a significant downregulation of GPR18, the RvD2 receptor^24^, as well as a trend towards the downregulation of PD1 receptor GPR37^25^ in intermediate monocytes and MaR1 receptor LGR6^26^ in all three monocytes subsets (Figure 1H-J). Together, these findings lend support to the hypothesis that resolution mechanisms are altered in peripheral blood phagocytes from COVID-19 patients.

### Peripheral blood phagocytes from COVID-19 patients display an altered activation status and function

**Figure 2:**
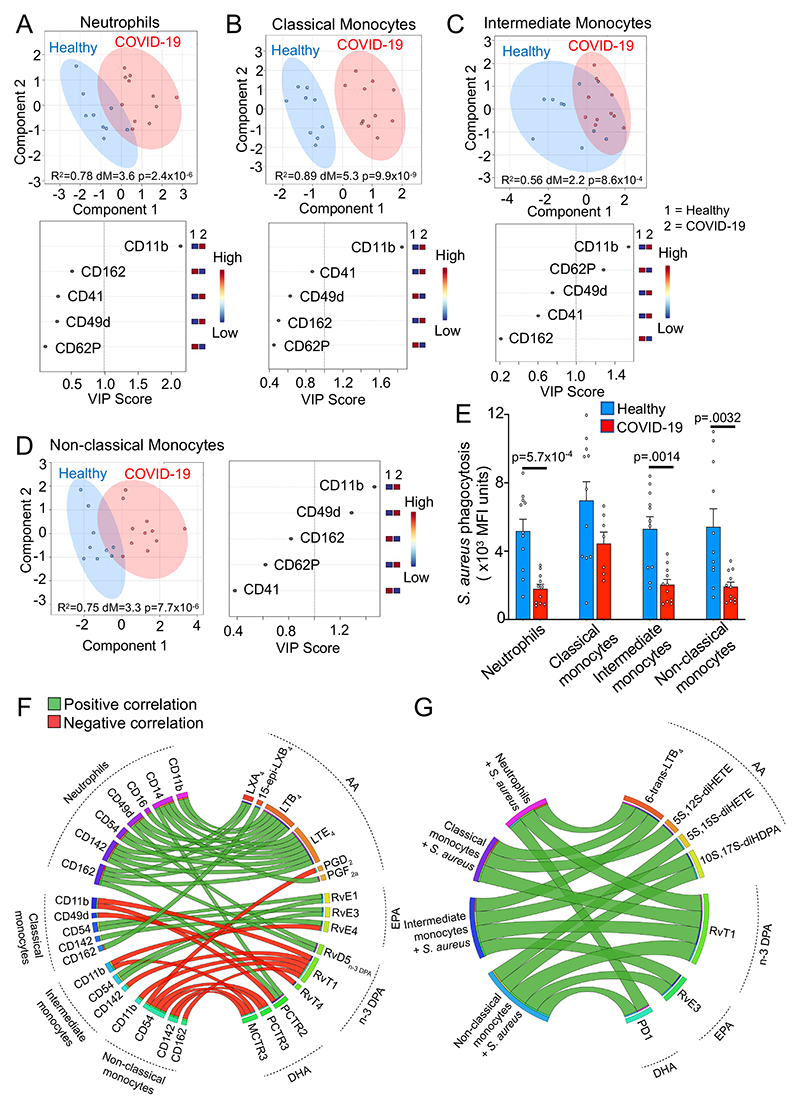
Upregulation of activation markers and downregulation of *S*. *aureus* phagocytosis in circulating phagocytes from COVID-19 patients. (**A-D**) PLS-DA scores plots with 95% CI and VIP scores for activation markers on circulating phagocytes from healthy volunteers (n=9) and COVID-19 patients (n=11) assessed by flow cytometry. (**E**) *S*. *aureus* phagocytosis by peripheral blood neutrophils and monocyte subsets from healthy volunteers (n=11) and COVID-19 patients (n=11) evaluated using flow cytometry. Statistical differences between Healthy and COVID-19 groups were established using Mann-Whitney Test for each cell type and raw p-values are displayed. (**F-G**) Circos plots of Pearson correlation coefficients between COVID-19 patient (n=11) plasma LM levels and (**F**) phagocyte activation markers or (**G**) bacterial phagocytosis. Connecting bands represent statistically significant correlations (p<0.05), with the width of the band being proportional to the strength of the correlation (range −0.66-0.83) and band colour representing positive (green) or negative (red) correlations.

We next assessed whether these changes in SPM pathways were reflected in a dysregulation of circulating phagocyte activation status and function. For this purpose, we evaluated the expression of proteins linked with the activation status of circulating phagocytes. PLS-DA unveiled an overall shift in the activation status of all four phagocyte subsets evaluated, as demonstrated by a separation of healthy volunteer and COVID-19 patient clusters (Figure 2A-D). In all phagocyte subsets, this phenotypic shift was primarily driven by upregulation of the integrin Cluster of Differentiation (CD)11b (Figure 2A-D). Additionally, we observed a downregulation in the expression of CD62P, a marker of platelet-monocyte heterotypic aggregates^19^ intermediate monocytes (Figure 2C). Whereas, in non-classical monocytes we observed a marked upregulation of the integrin CD49d (Figure 2D).

Several studies found that patients with COVID-19 pneumonia may also develop secondary bacterial infections^27, 28^. Thus, we next evaluated whether phagocytes from COVID-19 patients displayed altered phagocytic function. We evaluated the ability of peripheral blood phagocytes to uptake and kill *Staphylococcus aureus* as an exemplar bacterium of clinical relevance using bacterial particles labelled with a pH-sensitive fluorophore to monitor uptake and phagolysosome acidification, a key step in bacterial killing. This analysis demonstrated a significant downregulation in fluorescence levels in neutrophils, intermediate monocytes and non-classical monocytes from COVID-19 patients incubated with fluorescently labelled *S. aureus* (Figure 2E).

To evaluate whether the variations in SPM concentrations observed in COVID-19 patients were linked to changes in peripheral blood phagocyte activation status and function, we next conducted a correlation analysis (Figure 2F). This analysis demonstrated that arachidonic acid (AA)-derived mediator levels, such as those of Leukotriene (LT)B_4_, and LTE_4_, were generally positively correlated with activation marker expression on neutrophils, while n-3 DPA and DHA-derived mediators such as MCTR3, PCTR3, and RvT1 were negatively correlated with expression of activation markers on all three monocytes subsets. MCTR3 and PCTR3 were also significantly negatively correlated with CD54 (ICAM-1) and CD142 (Tissue factor) expression on non-classical monocytes, while RvT1 was negatively correlated with expression of integrin CD11b on classical and non-classical monocytes (Figure 2F). Plasma SPM concentrations were also linked with the ability of different phagocyte subsets to uptake bacteria. Here, we observed a significant positive correlation between plasma PD1, RvT1, RvE3 and 10S, 17S-diHDPA and the ability of neutrophils and monocytes to uptake *S*. *aureus* (Figure 2G). Taken together, these findings suggest that changes in SPM pathways are linked with an alteration in phagocyte activation status and function in COVID-19 patients.

### Downregulation of SPM in patients with severe COVID-19 pneumonia

**Figure 3:**
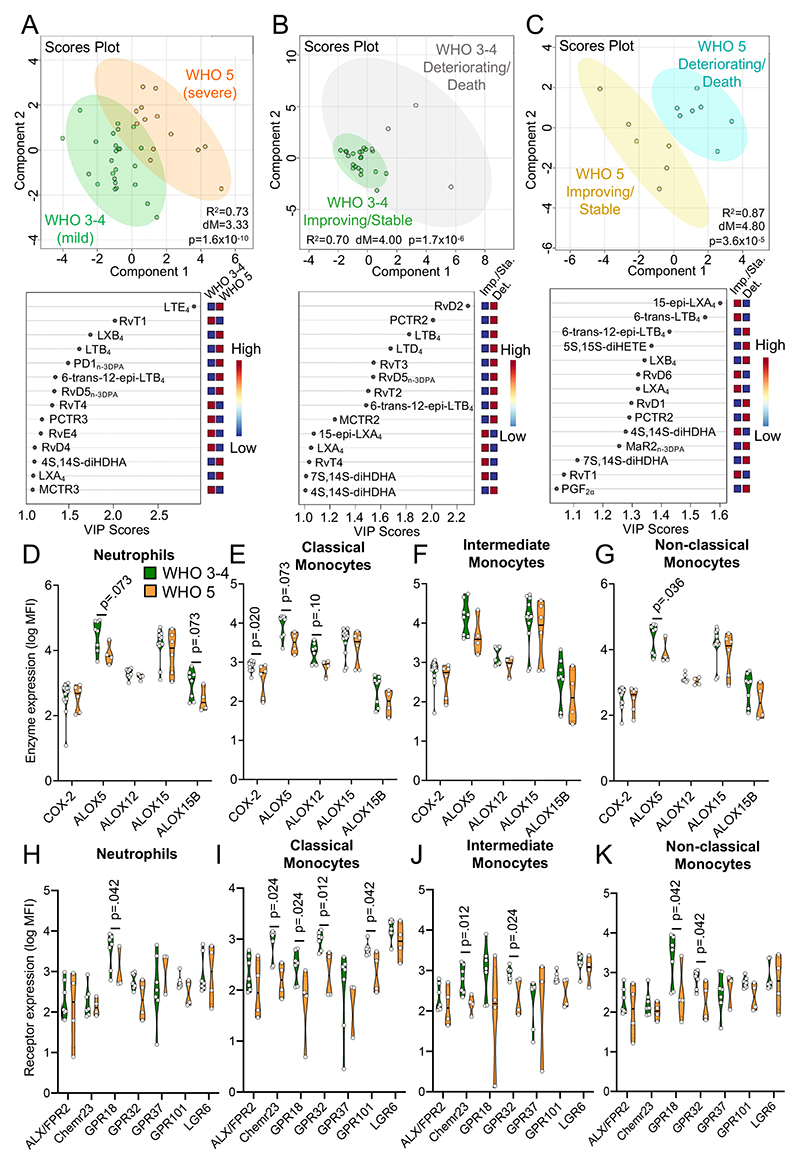
Disruption of SPM pathways with increased disease severity in COVID-19 patients. Peripheral blood was collected from COVID-19 patients with mild (WHO scale 3-4; WHO 3-4) or severe disease (WHO scale 5; WHO 5). (**A-C**) Plasma lipid mediators were identified and quantified using LC-MS/MS based lipid mediator profiling and PLS-DA was performed on identified mediators for the indicated patient sub-groups. *Top panel* – Scores plot with 95% CI. *Bottom Panel* – VIP scores plot. For (**A**), n=25 WHO 3-4 patients and n=13 WHO 5 patients; for (**B**), n=21 WHO 3-4 improving/stable patients and n=4 WHO 3-4 deteriorating/death patients; for (**C**), n=6 WHO 5 improving/stable patients and n=7 WHO 5 deteriorating/death patients. (**D-K**) Whole blood from COVID-19 patients was incubated with lineage-marker antibodies for neutrophils and monocyte subsets in combination with antibodies against (**D-G**) lipid mediator biosynthetic enzymes or (**H-K**) SPM receptors and their expression was evaluated using flow cytometry. For (**D-G**), n=14 WHO 3-4 patients (for COX-2, ALOX15), n=8 WHO 3-4 patients (for ALOX5, ALOX12, ALOX15B), n=6 WHO 5 patients (for COX-2, ALOX15), n=6 WHO 5 patients (for ALOX5, ALOX12, ALOX15B). For (**H-K**), n=7 WHO 3-4 patients and n=4 WHO 5 patients (except for GPR37 where n=3 WHO 5 patients). Statistical differences between WHO 3-4 and WHO 5 COVID-19 patients were established using Mann-Whitney Test for each molecule and raw p-values are displayed.

We next investigated whether plasma LM concentrations differed with increasing disease severity in COVID-19 patients. For this purpose, we assessed plasma LM concentration in hospitalized patients with mild disease and compared them with those with severe disease based on their WHO Ordinal Scale of COVID-19 Disease Severity at time of sample collection. Using PLS-DA we found a marked shift in plasma LM concentrations in patients with a WHO scale of 3 or 4 (WHO 3-4), which indicates hospitalised patients with mild disease symptoms, when compared to those with a WHO scale of 5 (WHO 5), indicative of more severe pneumonia requiring non-invasive ventilation or high-flow oxygen (see Supplemental Table III for clinical characteristics). This shift in plasma LM profiles was due to an overall downregulation in DHA-derived SPM such as PCTR3 and MCTR3 in patients with severe disease, which was coupled with an upregulation of AA-derived LM, including the potent leukocyte chemoattractant LTB4 and LTE4, the downstream product of the pro-inflammatory smooth muscle-contractants LTC_4_ and LTD_4_ (Figure 3A and Supplemental Table IV).

To investigate the impact of disease severity on LM profiles in more detail, we evaluated LM profiles in patients with mild (WHO 3-4) and severe disease (WHO 5) according to their disease trajectory. Here, we separated patients based on whether their symptoms were improving or stable versus those that were either clinically deteriorating at time of sample collection or would ultimately succumb to the disease (referred to as *deteriorating;* Figure 3B-C, Supplemental Tables III, VII, VIII). PLS-DA demonstrated a shift in LM profiles between clinically improving/stable patients compared to deteriorating patients in patients with mild disease (Figure 3B). Notably, this distinction in LM profiles was observed to be even more pronounced in patients with severe disease (Figure 3C). For patients with mild disease, differences between improving/stable and deteriorating groups were primarily linked with higher concentrations of both SPM (i.e. RvD2, PCTR2, RvT2, RvT3, and RvD5_n-3 DPA_) and leukotrienes (i.e. LTB4 and LTD4) in patients that were clinically deteriorating (Figure 3B). On the other hand, differences between these two groups in patients with severe disease were primarily driven by higher levels of pro-resolving 15-epi-LXA_4_, RvD6, LXA_4_ and RvT1 in patients that were clinically improving or stable (Figure 3C).

To explore the mechanisms underlying the altered SPM production in severe COVID-19 patients, we analysed the biosynthetic pathways of mediators with a VIP score >1 in the PLS-DA (shown in Figure 3A). This analysis identified a downregulation in the levels of DHA and EPA-derived ALOX5-ALOX15 interaction products, including RvD4 and RvE4. A reduction that was coupled with an increase in LXA_4_ and LXB_4_ concentrations, the AA-derived products from these enzymes, in patients at WHO scale 5 (Supplemental Figure I-B). We also observed a decrease in peptide-lipid conjugated SPM (PCTR3 and MCTR3), suggesting that there was a reduction in activity of either the initiating ALOX enzymes in each of these pathways (ALOX15 or ALOX12, respectively) or their shared downstream biosynthetic enzymes. Notably, these downstream biosynthetic enzymes also produce cystenyl-leukotrienes such as LTE_4_, which was increased in patients with severe disease, suggesting a shift in the activity of these enzymes towards the formation of pro-inflammatory mediators (Supplemental Figure I-B).

We next assessed the expression of SPM biosynthetic enzymes in circulating phagocytes. A trend for reduced expression of all enzymes under analysis with increased disease severity could be observed across all four phagocyte subsets, which reached statistical significance for ALOX5 and ALOX15B in neutrophils, COX-2 and ALOX5 in classical monocytes, and ALOX5 in non-classical monocytes (Figure 3D-G). These results suggest that the differential regulation of SPM production observed in plasma from severe patients was in part linked with a downregulation of SPM biosynthetic enzyme expression in circulating phagocytes.

We next evaluated whether SPM receptor expression was also altered with increased disease severity. Here, we found reduced expression of multiple SPM receptors on circulating phagocytes from severe COVID-19 patients. This included a significant downregulation of GPR18 on neutrophils, classical monocytes, and non-classical monocytes, as well as reduced expression of ChemR23, the receptor for RvE1 and RvE2, on classical and intermediate monocytes. We also observed a significant downregulation in GPR32 on intermediate and non-classical monocytes, and a decrease in GPR101 on classical monocytes in patients with severe disease (Figure 3H-K). Together, these findings suggest that resolution pathways become dysregulated in COVID-19 patients with severe disease.

### Altered resolution pathways persist after resolution of COVID-19 clinical symptoms

**Figure 4:**
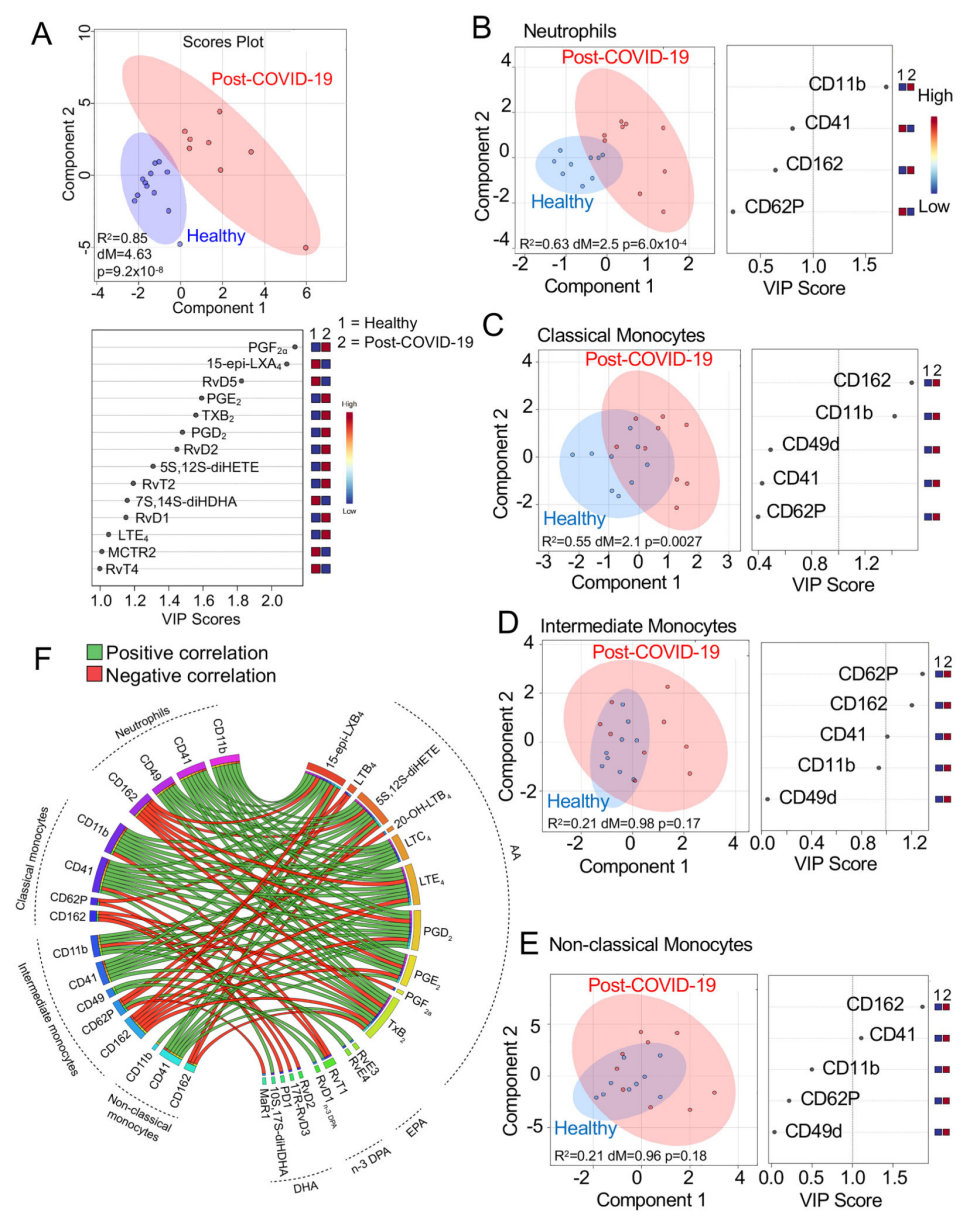
SPM pathways remain dysregulated after the resolution of clinical symptoms in SARS-CoV-2 infected patients. Peripheral blood was collected from volunteers after the resolution of COVID-19 clinical symptoms (post-COVID-19) or healthy volunteers. (**A**) Plasma lipid mediators were identified and quantified using LC-MS/MS based lipid mediator profiling and PLS-DA was performed on identified mediators (n=12 healthy volunteers, n=8 post-COVID-19 volunteers). *Top panel* – Scores plot with 95% CI. *Bottom Panel* – VIP scores plot. (**B-E**) PLS-DA scores plots and VIP scores for activation markers on circulating phagocytes assessed by flow cytometry of peripheral blood from healthy (n=9) and post-COVID-19 (n=8) volunteers. (**F**) Circos plots of Pearson correlation coefficients between post-COVID-19 volunteer (n=8) plasma LM levels and circulating phagocyte activation markers. Connecting bands represent statistically significant correlations (p<0.05), with the width of the band being proportional to the strength of the correlation (range −0.93-0.93) and band colour representing positive (green) or negative (red) correlations.

Having observed a marked shift in LM profiles from patients with active COVID-19 pneumonia, we next evaluated whether plasma LM profiles returned to baseline after the resolution of COVID-19 clinical symptoms (referred to as post-COVID-19). We collected peripheral blood from post-COVID-19 volunteers between 12-25 days after the resolution of clinical symptoms (n=8) and compared their plasma LM profiles with those obtained from healthy volunteers with no clinical suggestion of COVID-19 infection (n=12; see Supplemental Table I for demographics). PLS-DA demonstrated that LM profiles obtained from post-COVID-19 volunteers were markedly different from those obtained from healthy volunteers (Figure 4A and Supplemental Table II). This separation was primarily driven by differential regulation of 14 mediators that included both pro-inflammatory eicosanoids, such as prostaglandin (PG)E2, PGF_2α_, and LTE_4_, and SPM, RvD1, RvD2 and RvT2 (Figure 4A).

Examination of LM biosynthetic pathways indicated an upregulation of COX activity in post-COVID-19 volunteers, as indicated by an increase in both pro-inflammatory (i.e. PGD_2_, PGE_2_ and PGF_2a_) and pro-resolving (RvT2) COX-derived products in these volunteers. We also found an overall increase in ALOX5-ALOX15 interaction products, including RvD1 and RvD2, and an increase in the ALOX5 products 5S, 12S-diHETE and LTE_4_ in post-COVID-19 volunteers when compared with healthy volunteers (Supplemental Figure II-A). Intriguingly, expression of these biosynthetic enzymes in circulating phagocytes form post-COVID-19 patients was essentially the same as that observed in healthy volunteers (Supplemental Figure II-C-F). Thus, these results suggest that the alterations in resolution pathways observed in COVID-19 patients can persist even after the subsidence of clinical symptoms, and might arise from changes in activity of the LM biosynthetic enzymes.

### Changes in peripheral blood phagocyte activation status partially persist after the resolution of COVID-19 clinical symptoms

We next assessed whether the observed changes in SPM pathways were linked with changes in peripheral blood phagocyte activation status and function in post-COVID-19 volunteers. Flow cytometric analysis demonstrated that post-COVID-19 neutrophils and classical monocytes displayed an activated phenotype as observed by marked shifts in the clusters representing these cells versus the clusters representing cells from healthy volunteers. This separation was primarily linked with an upregulation in CD11b on these cells (Figure 4B-C), an observation also previously made with cells from COVID-19 pneumonia patients (Figure 2A-D). Conversely, the activation status of intermediate and non-classical monocyte from post-COVID-19 volunteers was not markedly different from healthy volunteer cells (Figure 4D-E). Comparison of phagocytic ability of cells isolated from post-COVID-19 volunteers demonstrated that phagocytes from these volunteers displayed an essentially similar ability to uptake fluorescently labelled *S*. *aureus* when compared with healthy volunteers (Supplemental Figure II-B). We also did not observe marked differences in SPM receptor expression on circulating phagocytes from post-COVD-19 volunteers when compared with healthy volunteers (Supplemental Figure II-G-J).

We next evaluated whether changes in phagocyte activation observed in post-COVID-19 volunteers were correlated with the observed changes in peripheral blood SPM concentrations (Figure 4F). Here we found that neutrophil and monocyte expression of the platelet-leukocyte heterotypic aggregate marker CD41 and integrin CD11b positively correlated with a number of AA-derived leukotrienes and prostaglandins, while the SPM 15-epi-LXB_4_, 17R-RvD3, and MaR1 negatively correlated with CD62P, another marker of leukocyte-platelet aggregates (Figure 4F). Taken together, these findings indicate that alterations in phagocyte activation status and peripheral LM profiles persist after the resolution of clinical symptoms in SARS-CoV-2 infected individuals.

### Dexamethasone upregulates peripheral blood SPM concentrations in COVID-19 patients

**Figure 5:**
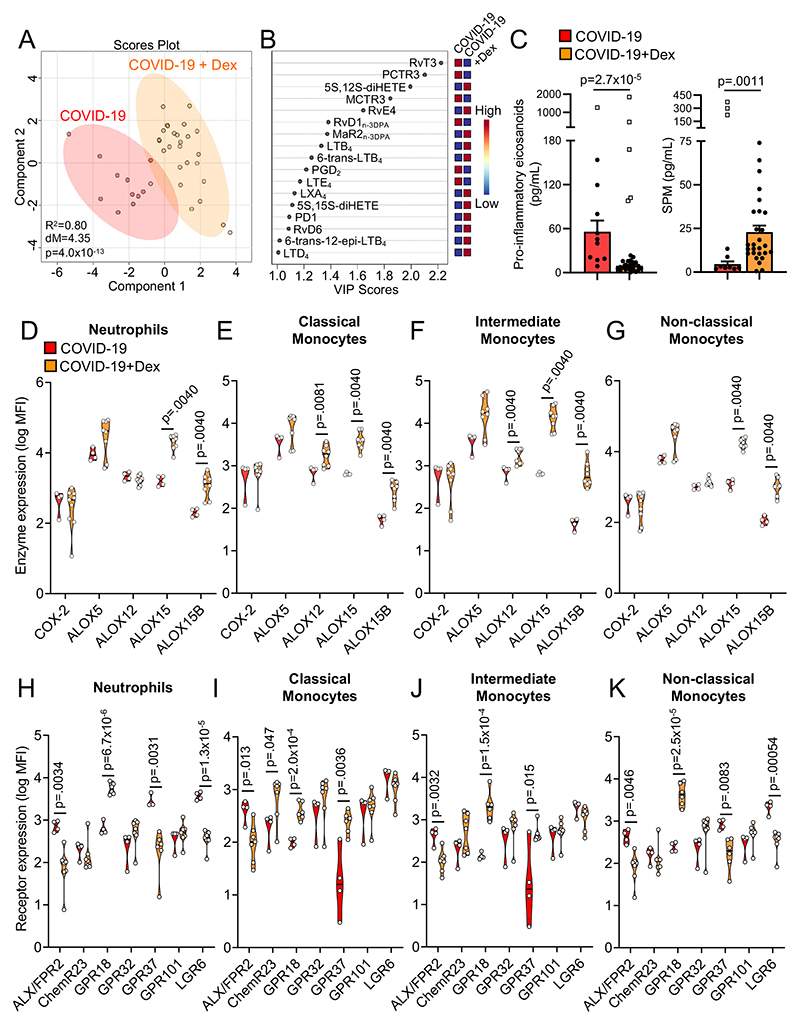
Dexamethasone upregulates plasma SPM in COVID-19 pneumonia patients. Peripheral blood was collected from COVID-19 patients treated with or without dexamethasone (Dex; 6mg/day). (**A-B**) Plasma lipid mediators were identified and quantified using LC-MS/MS based lipid mediator profiling. PLS-DA was performed on identified mediators, (**A**) PLS-DA Scores Plot with 95% CI and (**B**) VIP scores plot. (**C**) *Left panel* - cumulative pro-inflammatory eicosanoids (PG, LT and TX); *Right panel* – cumulative SPM (Rv, PD, MaR, LX). Open squares indicate samples identified as statistical outliers using ROUT test (Q=0.2%) and statistical differences were established using Mann-Whitney Test (for which outliers were excluded). n=11 COVID-19 and n=27 COVID-19+Dex patients. (**D-K**) Blood was collected from COVID-19 or COVID-19+Dex patients and incubated with lineage-marker antibodies for neutrophils and monocyte subsets in combination with antibodies against (**D-G**) lipid mediator biosynthetic enzymes or (**H-K**) SPM receptors and their expression was evaluated using flow cytometry. For (**D-G**), n=4 COVID-19 patients and n=8 COVID-19+Dex patients (except for COX-2 where n=9 COVID-19+Dex patients). For (**H-K**), n=4 COVID-19 patients and n=7 COVID-19+Dex patients (except for GPR18 and GPR37 where n=6 COVID-19+Dex patients). Statistical differences between COVID-19 and COVID-19+Dex patients were established using Mann-Whitney Test for each molecule and raw p-values are displayed.

Recent studies demonstrate that dexamethasone upregulates SPM formation in experimental allergic inflammation^29^. Therefore, we questioned whether this corticosteroid also regulated SPM formation in COVID-19 patients. Plasma LM concentrations were measured in COVID-19 patients treated with or without 6mg/day of dexamethasone (see Supplemental Table V for patient information). Analysis of plamsa LM profiles using PLS-DA demonstrated a distinct clustering of the groups representing patients that received dexamethasone (COVID-19+Dex) versus those that did not (COVID-19; Figure 5A,B and Supplemental Table VI). This shift in plasma lipid mediator profiles was linked with an overall downregulation in plasma pro-inflammatory eicosanoid concentrations and an upregulation of plasma SPM concentrations (Figure 5C).

Dexamethasone is known to regulate SPM enzyme expression in experimental settings^30^. We therefore evaluated whether the expression of SPM enzymes in circulating phagocytes was affected by dexamethasone treatment. Flow cytometric evaluation demonstrated a significant upregulation of ALOX15 and ALOX15B in all phagocyte subsets, as well as ALOX12 in classical and intermediate monocytes of COVID-19 patients treated with dexamethasone (Figure 5D-G). These findings suggest that dexamethasone increases peripheral blood SPM concentration in COVID-19 patients *via* the upregulation of SPM biosynthetic enzymes in peripheral blood phagocytes.

We next explored whether dexamethasone also regulated the expression of SPM receptors on circulating phagocytes. Flow cytometric analysis demonstrated that while expression of ALX/FPR2 (the receptor for LXA4, RvD1, RvD3 and their aspirin-triggered epimers) was significantly downregulated on all four phagocyte subsets, the expression of GPR18 was upregulated on these cells (Figure 5H-K). Moreover, GPR37 was downregulated on neutrophils and non-classical monocytes whilst it was upregulated on classical and intermediate monocytes (Figure 5H-K). Together these findings demonstrate that in addition to upregulating plasma SPM concentrations, dexamethasone also regulates SPM receptor expression on circulating phagocytes from COVID-19 patients.

### SPM rectify peripheral blood phagocyte responses

**Figure 6:**
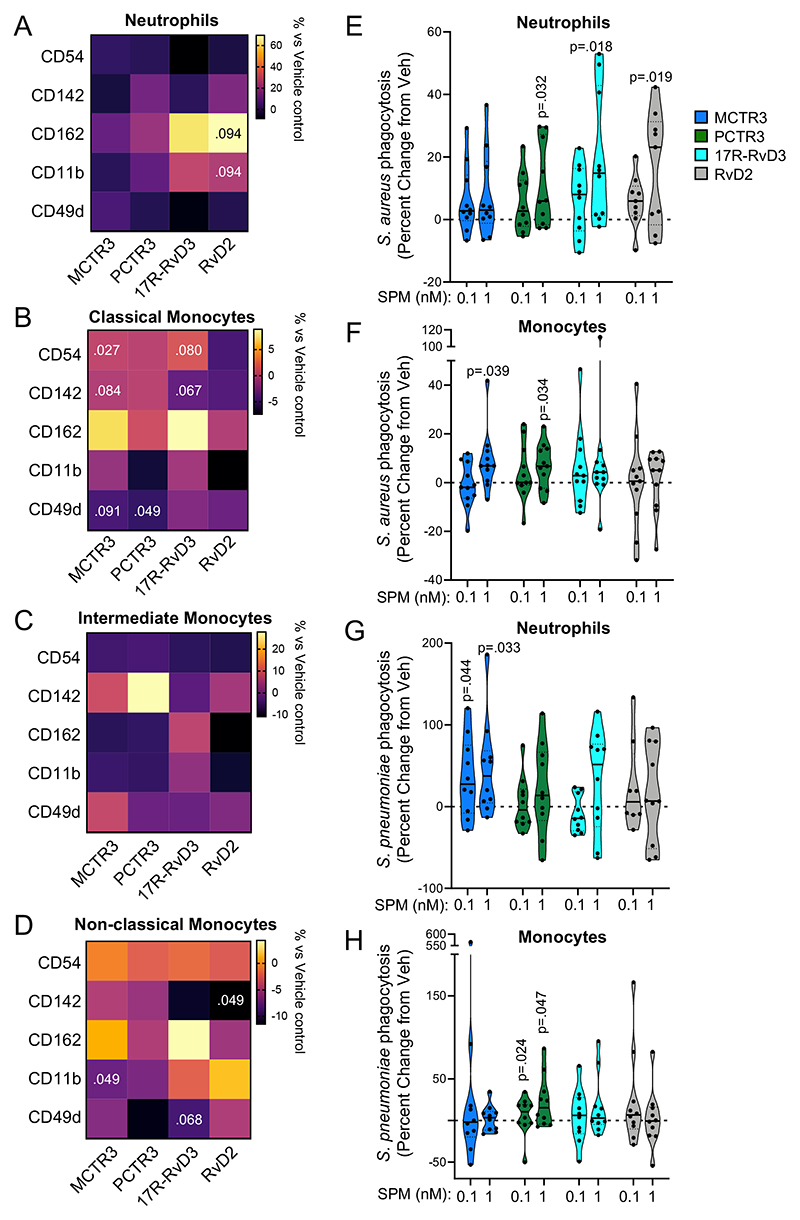
SPM regulate COVID-19 phagocyte activation and bacterial phagocytosis. Neutrophils and monocytes were isolated from peripheral blood of COVID-19 patients using density centrifugation. (**A-D**) Cells were incubated with 1nM of indicated SPM or vehicle for 60 minutes and expression of adhesion molecules was assessed using flow cytometry. Results are reported as percentage change from expression levels in cells incubated with vehicle only. Statistical differences were established using Wilcoxon Signed Rank test for each molecule and raw p-values are displayed. n=10 for neutrophils and n=17 for monocytes. (**E-H**) Neutrophils (**E,G**) and monocytes (**F,H**) were incubated with indicated SPM or vehicle for 15 minutes followed by fluorescently-labelled (**E,F**) *S*. *aureus* or (**G,H**) *S pneumoniae* and phagocytosis assessed in real-time using high-content imaging. Results are expressed as percent change from fluorescence levels recorded in cells incubated with vehicle only. For (**E,G**), n=10 COVID-19 patients (except RvD2 0.1nM where n=8). For (**F**), n=16 COVID-19 patients. For (**H**), n=12 COVID-19 patients. Statistical differences between SPM treatments and vehicle were established using Wilcoxon Signed Rank Test for each mediator and raw p-values are displayed.

SPM potently regulate phagocyte activation status as well as their ability to uptake and kill bacteria^13, 21, 24, 26, 31, 32^. Having observed that dexamethasone upregulated plasma SPM concentrations we next evaluated the translational potential of these findings by testing the pharmacological properties of specific SPM in regulating the observed alterations in peripheral blood phagocytes. For this purpose, we focused on MCTR3, PCTR3, 17R-RvD3, and RvD2, given that plasma concentrations of MCTR3 and PCTR3 were found to correlate with phagocyte activation in COVID-19 patients and their levels were decreased in patients with severe disease. Whereas the receptors for 17R-RvD3 (GPR32), and RvD2 (GPR18) were differentially regulated on phagocytes from these patients. Here we found that each SPM tested displayed characteristic regulatory activities on the four phagocyte subsets (Figure 6). While these mediators did not significantly alter expression of activation markers on neutrophils (Figure 6A), MCTR3 treatment significantly decreased CD54, while PCTR3 downregulated CD49d expression on classical monocytes (Figure 6B). MCTR3 also decreased CD11b expression on non-classical monocytes (Figure 6D). Recent studies found that expression of the coagulation-initiating CD142 is upregulated on circulating monocytes from COVID-19 patients, an increase that was linked with enhanced disease severity and mortality^33^. Incubation of monocytes with all four SPM tested led to an overall downregulation of CD142, reaching statistical significance for RvD2 treatment of non-classical monocytes (Figure 6B-D).

Evaluation of the ability of MCTR3, PCTR3, 17R-RvD3, and RvD2 to regulate phagocyte responses based on disease severity (i.e. WHO ordinal scale), demonstrated a trend towards upregulation of CD162 on neutrophils from COVID-19 patients with milder disease. Whereas, these SPM did not regulate neutrophil activation markers on cells from patients with more severe disease (WHO scale 5; Supplemental Figure III-A). The same outcome emerged when monocytes were analysed, with SPM being more effective on classical and intermediate monocytes of WHO scale 3-4 patients (Supplemental Figure III-B-D). Non-classical monocytes were the only phagocyte subset tested where the activity of SPM on the regulation of activation marker expression appeared to be higher in cells from patients with severe disease (Supplemental Figure III-D).

We next evaluated whether SPM rescued the defect in *S*. *aureus* phagocytosis observed in peripheral blood leukocytes isolated from COVID-19 patients. Incubation of all four SPM with peripheral blood neutrophils led to an increase in phagocytosis by neutrophils, which reached statistical significance for PCTR3, 17R-RvD3, and RvD2 at 1nM (Figure 6E). We observed increases in *S. aureus* phagocytosis by peripheral blood monocytes when these cells were incubated with 1nM of either MCTR3 or PCTR3 (Figure 6F). These mediators also significantly increased phagocytosis of *Streptococcus pneumoniae*, another clinically relevant bacterium in COVID-19^34^, by peripheral blood neutrophils and monocytes from COVID-19 patients, respectively (Figure 6G-H).

### SPM reprogram monocyte-derived macrophages from COVID-19 patients

**Figure 7:**
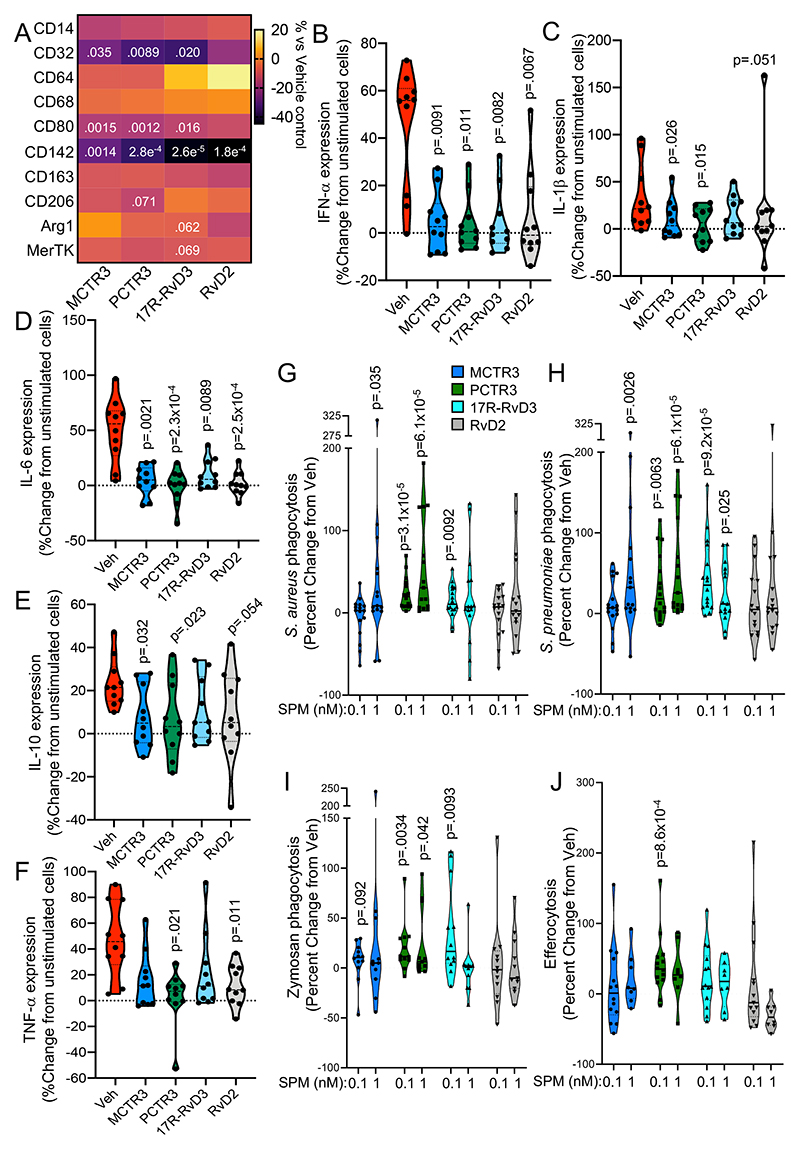
Regulation of COVID-19 monocyte-derived macrophage phenotype and function by SPM. (**A-F**) Monocytes were isolated from peripheral blood of COVID-19 patients and differentiated to monocyte-derived macrophages with GM-CSF in the presence of vehicle or 10nM of the indicated SPM. On day 7, (**A**) cells were lifted and the expression of the indicated phenotypic markers was assessed using flow cytometry (n=13 COVID-19 patients) or (**B-F**) cells were incubated with recombinant human S100A8/A9 dimer (1 μg/ml, 24 hours) and Brefeldin A (2 μg/ml, for final 18 hours) and the expression of indicated cytokines was assessed using flow cytometry (n=12 COVID-19 patients). Results are reported as percentage change in expression from levels in cells incubated with vehicle only. Statistical differences between vehicle and SPM treatments were established using Kruskal-Wallis test with Dunn’s post-hoc correction. (**G-I**) Monocytes from COVID-19 patients were differentiated to monocyte-derived macrophages with GM-CSF, incubated with the indicated SPM (0.1 or 1 nM) or vehicle for 15 minutes followed by fluorescently-labelled (**G**) *S*. *aureus*, (**H**) *S. pneumoniae*, (**I**) zymosan, or (**J**) apoptotic cells. Results are reported as percentage change in uptake from levels in cells incubated with vehicle only. For (**G-H**), n=16 (for 0.1nM SPM) and n=15 (for 1nM SPM) COVID-19 patients. For (**I**), n=12 (for 0.1nM SPM) and n=11 (for 1nM SPM) COVID-19 patients. For (**J**), n=14 (for 0.1nM SPM) and n=8 (for 1nM SPM) COVID-19 patients. Statistical differences between SPM treatments and vehicle were established using Wilcoxon Signed Rank Test for each mediator and raw p-values are displayed.

Monocyte-derived macrophages are linked with onset, progression and resolution of both acute and chronic inflammation^4, 13, 35^. Furthermore, macrophage responses in COVID-19 patients are dysregulated, with these cells displaying an inflammatory phenotype that is linked with increased tissue inflammation and damage^12^. Having found that SPM regulate both monocyte activation and function, we next assessed whether these protective activities extended to monocyte-derived macrophages. For this purpose, we incubated monocytes isolated from peripheral blood of COVID-19 patients with SPM, differentiated them to macrophages using published protocols^23^, and assessed the expression of macrophage phenotypic markers using flow cytometry. Here we found that MCTR3, PCTR3, and 17R-RvD3 significantly downregulated CD32 and CD80 expression, whereas PCTR3 downregulated CD206 expression and 17R-RvD3 downregulated Arginase (Arg)-1 and MerTK expression. Notably, all four SPM tested significantly downregulated the expression of CD142 (Figure 7A). Furthermore, the activity of these SPM was found to be comparable in patients with mild and severe disease (Supplemental Figure IV).

The excessive production of pro-inflammatory cytokines, such as interleukin (IL)-1β, IL-6 and tumor necrosis factor (TNF)-α, is one of the hallmarks of severe COVID-19^9^. Macrophage-derived cytokines play a central role in the propagation of tissue inflammation. Thus, we next evaluated whether SPM regulated cytokine production in monocyte-derived macrophages. Incubation of these cells with S100A8/A9 dimer, a potent pro-inflammatory signalling molecule that is abundant in inflamed tissues from COVID-19 patients^8, 36, 37^, upregulated the expression of interferon (IFN)-α, IL-1β, IL-6, IL-10 and TNF-α (Figure 7B-F). Incubating macrophages with MCTR3, PCTR3, 17R-RvD3 or RvD2 led to a significant decrease in the expression of S100A8/A9-induced IFN-α, IL-1β, and IL-6 expression, while MCTR3, PCTR3 and RvD2 also significantly decreased expression of IL-10 and TNF-α (Figure 7B-F). Of note, assessment of cytokine expression when separating COVID-19 patients by disease severity based on WHO ordinal scale demonstrated that all four SPM were equally effective at reducing expression of these cytokines in cells from both patient groups (Supplemental Figure V).

We next assessed whether SPM regulated phagocytic responses in these cells. Incubation of monocyte-derived macrophages with MCTR3, PCTR3, or 17R-RvD3 increased their ability to clear the Gram-positive bacteria *S*. *aureus* and *S. pneumoniae* in a concentration-dependent manner (Figure 7G-H). Given that secondary infections by fungi from the *Aspergillus* genus are also common in COVID-19 patients^38^ we next tested whether these SPM also regulated the clearance of fungi. Here we found that MCTR3, PCTR3 and 17R-RvD3 dose-dependently increased uptake of fluorescently labelled zymosan, a fungal cell wall component (Figure 7I). Notably, while PCTR3 and 17R-RvD3 increased phagocytosis of *S. aureus, S. pneumoniae* and zymosan particles to a similar extent in cells from patients with mild disease when compared to those with severe disease, MCTR3 displayed greater activity with cells from patients with mild disease (Supplemental Figure VI).

Since the extensive and unresolved inflammation observed in the lungs of COVID-19 patients gives rise to widespread tissue damage and apoptosis, we next assessed the ability of SPM to enhance the clearance of apoptotic cells (a process termed efferocytosis). Here, we found that PCTR3 increased efferocytosis of apoptotic cells by monocyte-derived macrophages from COVID-19 patients in a concentration-dependent manner (Figure 7J). Together these findings demonstrate that SPM alter the function of monocyte-derived macrophages from COVID-19 patients towards a host protective phenotype.

## Discussion

Our study presents evidence that selective determinants of pro-resolving pathways are present in the blood of patients with COVID-19 pneumonia and suggests that their dysregulation, or improper engagement, may contribute to disease propagation. In peripheral blood from these patients, we observed an upregulation of SPM concentrations and expression of pro-resolving receptors on circulating phagocytes when compared with healthy volunteers, which became dysregulated with increasing disease severity. These changes were linked with altered neutrophil and monocyte activation status and function when compared to phagocytes from healthy volunteers. Treatment of COVID-19 patients with dexamethasone upregulated both peripheral blood SPM concentrations and the expression of their biosynthetic enzymes, while incubation of peripheral blood phagocytes with SPM modulated both their activation status and ability to uptake bacteria. In addition, SPM potently tempered monocyte-derived macrophage responses, downregulating the expression of pro-inflammatory cytokines and upregulating their ability to clear pathogenic microbes and apoptotic cells.

Phagocytes form the first line of defence against invading pathogens^39–41^. The behaviour of these cells is regulated by environmental cues that include soluble mediators such as cytokines and LM. Mounting evidence demonstrates that phagocyte behaviour becomes dysregulated in COVID-19 pneumonia patients with severe disease, contributing to uncontrolled systemic inflammation with ensuing organ damage^7, 33, 42–44^. Furthermore, these disrupted phagocyte responses are linked with an increased susceptibility to secondary infections^27, 28^. In the present study, we observed that overall lipid mediator concentrations are upregulated in plasma from COVID-19 pneumonia patients. This concomitant increase in both pro-inflammatory and pro-resolving mediators is a feature of several inflammatory conditions, including sepsis and acute respiratory distress syndrome^18^, and reflects the activation of counter-regulatory mechanisms by the host as an attempt to control unabated inflammation. Of note, Schwartz and colleagues reported that an increase in peripheral blood lipid mediator production is retained in serum from COVID-19 pneumonia patients^45^. The upregulation of SPM in both plasma and serum, together with an increase in the expression of SPM enzymes in peripheral blood monocyte subsets supports the hypothesis that activation of these cells is at least in part responsible for the observed increases in the concentrations of these molecules. Notably, plasma concentrations of SPM were decreased in those patients with more severe disease suggesting that inability of the host to upregulate these molecules leads to disease propagation. This hypothesis is further supported by the observation that in patients treated with dexamethasone, which has been shown to decrease disease severity and mortality in patients with severe COVID-19^6^, we observed increased plasma SPM concentrations as well as SPM biosynthetic enzyme expression in circulating phagocytes.

Recent studies demonstrate that the increased platelet-leukocytes heterotypic aggregates found in COVID-19 patients are linked with an upregulation of CD142 on monocytes^33^. CD142 initiates the extrinsic coagulation cascade, is fully functional when expressed on cell surfaces, and increased CD142 expression is linked with a state of hypercoagulability and elevated disease severity in patients with COVID-19 pneumonia^33^. Studies investigating the expression of CD142 indicate that the expression of this protein is regulated by both pro-inflammatory cytokines, such as IL-6^10^, and pro-inflammatory mediators such as thromboxane A_2_^46, 47^. In our studies we found that SPM downregulated the expression of this protein on all three monocyte subsets as well as on monocyte-derived macrophages. The regulation of this molecule by SPM was also observed to be comparable between cells from hospitalized patients with mild and severe disease. These results suggest that in addition to regulating leukocyte recruitment and function, SPM may also contribute to reducing the hypercoagulable state observed in COVID-19 patients by downregulating the expression of CD142 on circulating and tissue-resident phagocytes.

During inflammation, monocytes are recruited into inflamed tissues where they differentiate to monocyte-derived macrophages that either contribute to the propagation of tissue inflammation or promote its resolution and tissue repair to facilitate the re-establishment of tissue function. Studies in COVID-19 patients as well as in non-human primates demonstrate a marked increase in monocyte-derived macrophages numbers in various tissues, including lung, intestine, kidney, and liver^7, 42, 43^. These cells can contribute to the disseminated inflammatory status observed in COVID-19 patients *via* the production of both inflammatory cytokines such as IL-6, TNF-α and IFNs, as well as immunosuppressive agents such as IL-10^11, 43^. In the present study, we found that SPM potently downregulated the production of several inflammatory cytokines by monocyte-derived macrophages from COVID-19 patients. Intriguingly, Type I interferons and PGE2 downregulate bacterial phagocytosis by alveolar macrophages^48, 49^, suggesting that the decreased bacterial phagocytosis by COVID-19 patient-derived phagocytes might be due to elevated levels of these pro-inflammatory molecules counteracting endogenous SPM signalling. Consistent with this hypothesis, incubation of patient-derived monocyte-derived macrophages with SPM decreased IFN-α expression and increased their ability to uptake bacteria. Reduced inflammatory cytokine expression was also linked with a downregulation in the expression of phenotypic markers, including CD80 and CD206, which were recently found to be upregulated on monocytes from COVID-19 patients that express higher levels of pro-inflammatory cytokines^44^.

At epithelial surfaces, monocyte-derived macrophages play a central role in the clearance of pathogens. Several studies found that COVID-19 patients may also develop secondary bacterial or fungal infections^27, 28^. Here we found that MCTR3, PCTR3 and 17R-RvD3 significantly upregulated the uptake and killing of both bacteria and fungi by macrophages, an activity that was comparable between cells from patients with mild and severe disease. Taken together, the present findings highlight the potential pharmacological utility of SPM in regulating COVID-19-related immune cell responses. Such SPM-based therapeutics could either involve the use of SPM analogues/mimetics or the enhancement of endogenous SPM production through the administration of SPM substrates and precursors. Of note, we recently found that this latter approach enhances circulating SPM levels in healthy individuals and patients with peripheral artery disease, while it tempered phagocyte phenotype and function^50, 51^.

The strengths of the present study are that it evaluates the correlation between changes in plasma SPM and changes in phagocyte functions in COVID-19 patients. Furthermore, it interrogates the potential pharmacological utility of SPM in rectifying altered phagocyte functions. There are also some limitations that should be considered when evaluating the present findings. First, we were unable to recruit individuals suffering from acute COVID-19 that did not require hospitalization (i.e. WHO scale 1-2). Nor were we able to obtain serial samples from the same patient over a period of time. Such patient groups and samples could provide further insight into when and how changes in LM profiles occur during COVID-19 disease progression. Additionally, our study was not sufficiently powered to explore potential interactions between COVID-19 and specific comorbidities known to affect LM concentrations in plasma. Notably, comparisons of LM concentrations in COVID-19 patients separated by disease severity (Figure 3) or dexamethasone treatment status (Figure 5) showed that there were no marked differences in the co-morbidities between these groups (Supplemental Tables III & V). Finally, future studies will be needed to establish the mechanisms that govern the observed changes in SPM biosynthesis. Here, we measured protein expression of key SPM biosynthetic enzymes as one aspect that defines their activity and therefore SPM production. For some of our comparisons, the changes in enzyme expression were consistent with changes observed in LM profiles (Figures 1, 3, 5). Intriguingly, we found that such a relationship was not observed between plasma SPM concentrations and enzyme expression in leukocytes from post-COVID-19 volunteers. This observation suggests that additional mechanisms, e.g. subcellular localization or post-translational modifications of the enzymes^52^, may contribute to the LM profiles obtained in plasma from post-COVID-19 volunteers (Supplemental Figure II).

In summation, in the present study we provide evidence that the host immune response engages resolution mechanisms as an attempt to limit the over-shooting inflammatory response following SARS-CoV-2 infection. However, these protective mechanisms appear to fail in patients with severe COVID-19 pneumonia, leading to systemic inflammation and dysregulated circulating phagocyte responses, which in turn contribute to secondary organ damage. Such inadequate resolution is linked with the downregulation of SPM receptors on leukocytes. Nonetheless, these receptors remain functional and incubation of phagocytes from COVID-19 patients with MCTR3, PCTR3, 17R-RvD3 or RvD2 rectified many of the phagocyte responses investigated. Together these findings shed new light on the mechanisms contributing to the disease propagation in COVID-19 pneumonia.

## Supplementary Material

Supplemental Materials

## Novelty and Significance

### What is known?

- Specialized pro-resolving mediators (SPM) are produced via enzymatic conversion of essential fatty acids and promote inflammatory resolution and tissue homeostasis.
- Failure to resolve inflammation in a timely manner leads to chronic inflammation.
- Severe cases of coronavirus disease 2019 (COVID-19) are characterized by a hyper-inflammatory state that is largely driven by innate immune cell dysfunction.

## Non-standard Abbreviations and Acronyms

AA: arachidonic acid
CD: cluster of differentiation
COVID-19: coronavirus disease 2019
DHA: docosahexaenoic acid
DPA: docosapentaenoic acid
EPA: eicosapentaenoic acid
IFN: interferon
IL: interleukin
LM: lipid mediator
MCTR: maresin conjugates in tissue regeneration
n-3: omega-3
PCTR: protectin conjugates in tissue regeneration
PD: protectins
PLS-DA: partial least-squares discriminant analysis
RvD: D-series resolvin
RvE: E-series resolvin
RvT: 13-series resolvin
SARS-CoV-2: severe acute respiratory syndrome coronavirus 2
SPM: specialized pro-resolving mediators
TNF: tumor necrosis factor

## References

[1] Gilroy DW, De Maeyer RPH, Tepper M, O’Brien A, Uddin M, Chen J, Goldstein DR, Akbar AN (2020). Treating exuberant, non-resolving inflammation in the lung; Implications for acute respiratory distress syndrome and COVID-19. Pharmacol Ther.

[2] Luissint AC, Parkos CA, Nusrat A (2016). Inflammation and the Intestinal Barrier: Leukocyte-Epithelial Cell Interactions, Cell Junction Remodeling, and Mucosal Repair. Gastroenterology.

[3] Robb CT, Regan KH, Dorward DA, Rossi AG (2016). Key mechanisms governing resolution of lung inflammation. Semin Immunopathol.

[4] Serhan CN, Levy BD (2018). Resolvins in inflammation: emergence of the pro-resolving superfamily of mediators. J Clin Invest.

[5] Poland GA, Ovsyannikova IG, Kennedy RB (2020). SARS-CoV-2 immunity: review and applications to phase 3 vaccine candidates. Lancet.

[6] Group RC, Horby P, Lim WS, Emberson JR, Mafham M, Bell JL, Linsell L, Staplin N, Brightling C, Ustianowski A, Elmahi E (2020). Dexamethasone in Hospitalized Patients with Covid-19 - Preliminary Report. N Engl J Med.

[7] Fahlberg MD, Blair RV, Doyle-Meyers LA, Midkiff CC, Zenere G, Russell-Lodrigue KE, Monjure CJ, Haupt EH, Penney TP, Lehmicke G, Threeton BM (2020). Cellular events of acute, resolving or progressive COVID-19 in SARS-CoV-2 infected non-human primates. Nat Commun.

[8] Guo Q, Zhao Y, Li J, Liu J, Yang X, Guo X, Kuang M, Xia H, Zhang Z, Cao L, Luo Y (2020). Induction of alarmin S100A8/A9 mediates activation of aberrant neutrophils in the pathogenesis of COVID-19. Cell Host Microbe.

[9] Kim JS, Lee JY, Yang JW, Lee KH, Effenberger M, Szpirt W, Kronbichler A, Shin JI (2021). Immunopathogenesis and treatment of cytokine storm in COVID-19. Theranostics.

[10] Loo J, Spittle DA, Newnham M (2021). COVID-19, immunothrombosis and venous thromboembolism: biological mechanisms. Thorax.

[11] Lu L, Zhang H, Dauphars DJ, He YW (2021). A Potential Role of Interleukin 10 in COVID-19 Pathogenesis. Trends Immunol.

[12] Merad M, Martin JC (2020). Pathological inflammation in patients with COVID-19: a key role for monocytes and macrophages. Nat Rev Immunol.

[13] Dalli J (2017). Does promoting resolution instead of inhibiting inflammation represent the new paradigm in treating infections?. Mol Aspects Med.

[14] Ramon S, Baker SF, Sahler JM, Kim N, Feldsott EA, Serhan CN, Martinez-Sobrido L, Topham DJ, Phipps RP (2014). The specialized proresolving mediator 17-HDHA enhances the antibody-mediated immune response against influenza virus: a new class of adjuvant?. J Immunol.

[15] Morita M, Kuba K, Ichikawa A, Nakayama M, Katahira J, Iwamoto R, Watanebe T, Sakabe S, Daidoji T, Nakamura S, Kadowaki A (2013). The lipid mediator protectin D1 inhibits influenza virus replication and improves severe influenza. Cell.

[16] Rajasagi NK, Reddy PB, Suryawanshi A, Mulik S, Gjorstrup P, Rouse BT (2011). Controlling herpes simplex virus-induced ocular inflammatory lesions with the lipid-derived mediator resolvin E1. J Immunol.

[17] Gomez EA, Colas RA, Souza PR, Hands R, Lewis MJ, Bessant C, Pitzalis C, Dalli J (2020). Blood pro-resolving mediators are linked with synovial pathology and are predictive of DMARD responsiveness in rheumatoid arthritis. Nat Commun.

[18] Dalli J, Colas RA, Quintana C, Barragan-Bradford D, Hurwitz S, Levy BD, Choi AM, Serhan CN, Baron RM (2017). Human Sepsis Eicosanoid and Proresolving Lipid Mediator Temporal Profiles: Correlations With Survival and Clinical Outcomes. Crit Care Med.

[19] Colas RA, Souza PR, Walker ME, Burton M, Zaslona Z, Curtis AM, Marques RM, Dalli J (2018). Impaired Production and Diurnal Regulation of Vascular RvDn-3 DPA Increase Systemic Inflammation and Cardiovascular Disease. Circ Res.

[20] Norris PC, Libreros S, Serhan CN (2019). Resolution metabolomes activated by hypoxic environment. Sci Adv.

[21] Chiang N, Serhan CN (2017). Structural elucidation and physiologic functions of specialized proresolving mediators and their receptors. Mol Aspects Med.

[22] Perretti M, Cooper D, Dalli J, Norling LV (2017). Immune resolution mechanisms in inflammatory arthritis. Nat Rev Rheumatol.

[23] Flak MB, Koenis DS, Sobrino A, Smith J, Pistorius K, Palmas F, Dalli J (2020). GPR101 mediates the pro-resolving actions of RvD5n-3 DPA in arthritis and infections. J Clin Invest.

[24] Chiang N, Dalli J, Colas RA, Serhan CN (2015). Identification of resolvin D2 receptor mediating resolution of infections and organ protection. J Exp Med.

[25] Bang S, Xie YK, Zhang ZJ, Wang Z, Xu ZZ, Ji RR (2018). GPR37 regulates macrophage phagocytosis and resolution of inflammatory pain. J Clin Invest.

[26] Chiang N, Libreros S, Norris PC, de la Rosa X, Serhan CN (2019). Maresin 1 activates LGR6 receptor promoting phagocyte immunoresolvent functions. J Clin Invest.

[27] Ripa M, Galli L, Poli A, Oltolini C, Spagnuolo V, Mastrangelo A, Muccini C, Monti G, De Luca G, Landoni G, Dagna L (2020). Secondary infections in patients hospitalized with COVID-19: incidence and predictive factors. Clin Microbiol Infect.

[28] Manohar P, Loh B, Nachimuthu R, Hua X, Welburn SC, Leptihn S (2020). Secondary Bacterial Infections in Patients With Viral Pneumonia. Front Med (Lausanne).

[29] Pyrillou K, Chairakaki AD, Tamvakopoulos C, Andreakos E (2018). Dexamethasone induces omega3-derived immunoresolvents driving resolution of allergic airway inflammation. J Allergy Clin Immunol.

[30] Colamorea T, Di Paola R, Macchia F, Guerrese MC, Tursi A, Butterfield JH, Caiaffa MF, Haeggstrom JZ, Macchia L (1999). 5-Lipoxygenase upregulation by dexamethasone in human mast cells. Biochem Biophys Res Commun.

[31] Chen J, Shetty S, Zhang P, Gao R, Hu Y, Wang S, Li Z, Fu J (2014). Aspirin-triggered resolvin D1 down-regulates inflammatory responses and protects against endotoxin-induced acute kidney injury. Toxicol Appl Pharmacol.

[32] Lee S, Nakahira K, Dalli J, Siempos II, Norris PC, Colas RA, Moon JS, Shinohara M, Hisata S, Howrylak JA, Suh GY (2017). NLRP3 Inflammasome Deficiency Protects against Microbial Sepsis via Increased Lipoxin B4 Synthesis. Am JRespir Crit Care Med.

[33] Hottz ED, Azevedo-Quintanilha IG, Palhinha L, Teixeira L, Barreto EA, Pao CRR, Righy C, Franco S, Souza TML, Kurtz P, Bozza FA (2020). Platelet activation and platelet-monocyte aggregate formation trigger tissue factor expression in patients with severe COVID-19. Blood.

[34] Ginsburg AS, Klugman KP (2020). COVID-19 pneumonia and the appropriate use of antibiotics. Lancet Glob Health.

[35] Motwani MP, Gilroy DW (2015). Macrophage development and polarization in chronic inflammation. Semin Immunol.

[36] Chen L, Long X, Xu Q, Tan J, Wang G, Cao Y, Wei J, Luo H, Zhu H, Huang L, Meng F (2020). Elevated serum levels of S100A8/A9 and HMGB1 at hospital admission are correlated with inferior clinical outcomes in COVID-19 patients. Cell Mol Immunol.

[37] Shaath H, Vishnubalaji R, Elkord E, Alajez NM (2020). Single-Cell Transcriptome Analysis Highlights a Role for Neutrophils and Inflammatory Macrophages in the Pathogenesis of Severe COVID-19. Cells.

[38] Alanio A, Delliere S, Fodil S, Bretagne S, Megarbane B (2020). Prevalence of putative invasive pulmonary aspergillosis in critically ill patients with COVID-19. LancetRespirMed.

[39] Campbell EL, Colgan SP (2015). Neutrophils and inflammatory metabolism in antimicrobial functions of the mucosa. J Leukoc Biol.

[40] Parkos CA (2016). Neutrophil-Epithelial Interactions: A Double-Edged Sword. Am J Pathol.

[41] Shalova IN, Lim JY, Chittezhath M, Zinkernagel AS, Beasley F, Hernandez-Jimenez E, Toledano V, Cubillos-Zapata C, Rapisarda A, Chen J, Duan K (2015). Human monocytes undergo functional reprogramming during sepsis mediated by hypoxia-inducible factor-1alpha. Immunity.

[42] Speranza E, Williamson BN, Feldmann F, Sturdevant GL, Perez LP, Meade-White K, Smith BJ, Lovaglio J, Martens C, Munster VJ, Okumura A (2021). Single-cell RNA sequencing reveals SARS-CoV-2 infection dynamics in lungs of African green monkeys. Sci Transl Med.

[43] Wang C, Xu J, Wang S, Pan S, Zhang J, Han Y, Huang M, Wu D, Yang Q, Yang X, Yang Y (2020). Imaging Mass Cytometric Analysis of Postmortem Tissues Reveals Dysregulated Immune Cell and Cytokine Responses in Multiple Organs of COVID-19 Patients. Front Microbiol.

[44] Zhang D, Guo R, Lei L, Liu H, Wang Y, Wang Y, Qian H, Dai T, Zhang T, Lai Y, Wang J (2020). COVID-19 infection induces readily detectable morphologic and inflammation-related phenotypic changes in peripheral blood monocytes. J Leukoc Biol.

[45] Schwarz B, Sharma L, Roberts L, Peng X, Bermejo S, Leighton I, Casanovas-Massana A, Minasyan M, Farhadian S, Ko AI, Yale IT (2021). Cutting Edge: Severe SARS-CoV-2 Infection in Humans Is Defined by a Shift in the Serum Lipidome, Resulting in Dysregulation of Eicosanoid Immune Mediators. J Immunol.

[46] Bode M, Mackman N (2014). Regulation of tissue factor gene expression in monocytes and endothelial cells: Thromboxane A2 as a new player. Vascul Pharmacol.

[47] Eilertsen KE, Osterud B (2002). The central role of thromboxane and platelet activating factor receptors in ex vivo regulation of endotoxin-induced monocyte tissue factor activity in human whole blood. J Endotoxin Res.

[48] Aronoff DM, Canetti C, Peters-Golden M (2004). Prostaglandin E2 inhibits alveolar macrophage phagocytosis through an E-prostanoid 2 receptor-mediated increase in intracellular cyclic AMP. J Immunol.

[49] Nakamura S, Davis KM, Weiser JN (2011). Synergistic stimulation of type I interferons during influenza virus coinfection promotes Streptococcus pneumoniae colonization in mice. J Clin Invest.

[50] Schaller MS, Chen M, Colas RA, Sorrentino TA, Lazar AA, Grenon SM, Dalli J, Conte MS (2020). Treatment With a Marine Oil Supplement Alters Lipid Mediators and Leukocyte Phenotype in Healthy Patients and Those With Peripheral Artery Disease. J Am Heart Assoc.

[51] Souza PR, Marques RM, Gomez EA, Colas RA, De Matteis R, Zak A, Patel M, Collier DJ, Dalli J (2020). Enriched Marine Oil Supplements Increase Peripheral Blood Specialized Pro-Resolving Mediators Concentrations and Reprogram Host Immune Responses: A Randomized Double-Blind Placebo-Controlled Study. Circ Res.

[52] Fredman G, Ozcan L, Spolitu S, Hellmann J, Spite M, Backs J, Tabas I (2014). Resolvin D1 limits 5-lipoxygenase nuclear localization and leukotriene B4 synthesis by inhibiting a calcium-activated kinase pathway. Proc Natl Acad Sci U S A.

[53] Gomez EA, Colas RA, Souza PR, Hands R, Lewis MJ, Bessant C, Pitzalis C, Dalli J (2020). Blood pro-resolving mediators are linked with synovial pathology and are predictive of DMARD responsiveness in rheumatoid arthritis. Nat Commun.

[54] Xia J, Psychogios N, Young N, Wishart DS (2009). MetaboAnalyst: a web server for metabolomic data analysis and interpretation. Nucleic Acids Res.

[55] Worley B, Halouska S, Powers R (2013). Utilities for quantifying separation in PCA/PLS-DA scores plots. Anal Biochem.

[56] Krzywinski M, Schein J, Birol I, Connors J, Gascoyne R, Horsman D, Jones SJ, Marra MA (2009). Circos: an information aesthetic for comparative genomics. Genome Res.

