# Supplementary material for: Disrupted resolution mechanisms favour altered phagocyte responses in COVID-19
Source: Circ Res. Author manuscript; Available in PMC 2021 Aug 9. (PMC8336787; doi:10.1161/CIRCRESAHA.121.319142)
Supplement: Supplemental Materials [file EMS130953-supplement-Supplemental_Materials.pdf]

SUPPLEMENTAL MATERIALS
Expanded Materials & Methods
Supplemental Tables I – IX
Supplemental Figures I – VI
References 53-56

EXPANDED MATERIALS AND METHODS

Participants and study approvals

Peripheral venous blood was collected from patients admitted with acute COVID-19 pneumonia to medical wards at Barts Health NHS Trust, London, UK, after informed consent was taken and patient demographics and clinical information relevant to their admission were collected by members of the direct care team (NHS HRA RES Ethics 19/SC/0361). Mapping of disease severity to the WHO Ordinal Scale of COVID-19 Disease Severity as well as classification into Improving, Stable, or Deteriorating clinical condition was performed at time of sample collection by the clinician on site, prior to any sample analysis being conducted. Peripheral venous blood from healthy and post-COVID-19 volunteers that displayed no clinical symptoms of acute illness, including no symptoms of COVID-19, and were not contact-isolating after a (suspected) SARS-CoV-2 exposure at the time was collected after informed consent (NHS HRA RES Ethics 17/EM/0447). None of the post-COVID-19 volunteers were hospitalized for COVID-19-related symptoms at any point prior to sample collection (i.e. a WHO Ordinal Scale of 2 was the maximum for any individual in this group).

Blood collection and processing

Peripheral venous blood was collected in 5ml K2-EDTA Vacutainers (BD Biosciences). An aliquot of blood was transferred to 2ml tubes for whole blood assays (see below). The remaining blood was transferred to 50ml tubes and centrifuged 20min at 120x*g* with medium brake to separate the plasma fraction. Plasma (1ml) was transferred to 15ml tubes on dry ice containing 4ml methanol with deuterated internal standards for targeted lipid mediator profiling (see below). Remaining plasma was removed and red blood cells (RBC) were sedimented by mixing in 10ml Ca^{2+}/Mg^{2+}-free PBS and 8ml 6% (w/v) dextran (molecular weight 425.000-575.000; Sigma) followed by incubation at room temperature for 20 min. The RBC-depleted upper layers were then transferred to 50ml tubes containing 7.5ml Histopaque-1077 (Sigma) and centrifuged for 30min at 400x*g* with no brake to isolate polymorphonuclear leukocyte (PMN) and peripheral blood mononuclear cell (PBMC) cell fractions. Remaining RBCs in the PMN fraction were lysed by incubating in 9 volumes of ice-cold ddH_2O for 30 sec followed by addition of 1 volume 10x Hank's Balanced Salt Solution (HBSS). Cell numbers in the PMN and PBMC fractions were counted using a hemacytometer.

Targeted lipid mediator (LM) profiling by LC-MS/MS

Plasma samples from COVID-19 patients (n=38; *n.b.* one COVID-19 patient plasma sample was lost due to a technical failure during sample extraction), healthy volunteers (n=12), and post-COVID-19 volunteers (n=8) were extracted and lipid mediators were identified and quantified as described[53]. In brief, plasma was collected by centrifugation as described above and immediately placed on dry ice in 4 volumes of ice-cold methanol containing deuterated internal standards (d_4-LTB_4, d_5-LXA_4, d_4-PGE_2, d_5-RvD2, d_5-MaR1, d_5-MaR2, d_5-RvD3, d_4-RvE1, d_5-17R-RvD1, d_5-LTC_4, d_5-LTD_4 and d_5-LTE_4) representing each chromatographic region of identified LM. Following protein precipitation (-20°C for a minimum of 45 min), supernatants were extracted on an ExtraHera instrument (Biotage) using solid-phase extraction with Isolute C18 500mg columns (Biotage). Methyl formate and methanol fractions were collected, brought to dryness and resuspended in phase (methanol/water, 1:1, vol/vol) for injection on a Shimadzu LC-20AD HPLC and a Shimadzu SIL-20AC autoinjector, paired with a QTrap 5500 or QTrap 6500+ (Sciex). In the analysis of mediators eluted in the methyl formate fraction, the QTRAP 5500 was operated in negative ion mode using a multiple reaction monitoring method. An Agilent Poroshell 120 EC-C18 column (100 mm × 4.6 mm × 2.7 μm) was kept at 50 °C and mediators eluted using a mobile phase consisting of methanol/water/acetic acid of 20:80:0.01 (vol/vol/vol) that was ramped to 50:50:0.01 (vol/vol/vol) over 0.5 min and then to 80:20:0.01 (vol/vol/vol) from 2 min to 11 min, maintained till 14.5 min and then rapidly ramped to 98:2:0.01 (vol/vol/vol) for the next 0.1 min. This was subsequently maintained at 98:2:0.01 (vol/vol/vol) for 5.4 min, and the flow rate was maintained at 0.5 ml/min. In the analysis of mediators eluted in the methanol fraction, the QTrap 6500+ was operated in positive ion mode using a multiple reaction monitoring method. An Agilent Poroshell 120 EC-C18 column (100 mm × 4.6 mm × 2.7 μm) was kept at 50 °C and mediators eluted using a mobile phase consisting of methanol/water/acetic acid 55:45:0.5 (vol:vol:vol) over 5 min, that was ramped to 80:20:0.5 (vol:vol:vol) for 2 min, maintained at 80:20:0.5 (vol:vol:vol) for the successive 3 min and ramped to 98:2:0.5 (vol:vol:vol) over 3 min. This condition was maintained for 3 min. Each

lipid mediator was identified using established criteria, including: (1) presence of a peak with a minimum area of 2000 counts, (2) matching retention time to synthetic or authentic standards, (3) ≥4 data points, and (4) matching of at least 6 diagnostic ions to that of reference standard, with a minimum of one backbone fragment being identified. Calibration curves were obtained for each mediator using synthetic compound mixtures at 0.78, 1.56, 3.12, 6.25, 12.5, 25, 50, 100, and 200 pg that gave linear calibration curves with R^2 values of 0.98–0.99. These calibration curves were then used to calculate the abundance of each mediator per 1mL of plasma for each individual sample. These values and the specific deuterated internal standard used for concentration calculations of each mediator are indicated in Supplemental Tables II, IV, VI, VIII. Limits of the assay were determined in charcoal stripped plasma and are displayed in Supplemental Table IX.

Whole blood assays

Bacterial particle phagocytosis by peripheral blood leukocytes

Escherichia coli and *Staphylococcus aureus* bacterial particles conjugated with pHrodo Red or pHrodo Green pH-sensitive fluorophores (Invitrogen) were opsonized by incubating in 20% (v/v) human serum (male A/B; Sigma) for 30 min at 37°C. Whole blood was transferred to 15ml tubes (50µl/experimental condition), 25µl opsonized pHrodo Red *E.coli* or pHrodo Green *S. aureus* bioparticles were added (5µg bioparticles/sample), and samples were incubated 45 min at 37°C to allow phagocytosis to proceed. Samples were centrifuged 5 min at 2000 x *g* and cell pellets were washed with Ca^{2+}/Mg^{2+}-free PBS containing 0.02% (w/v) bovine serum albumin. Samples were then stained with fluorophore-conjugated antibodies against human CD16 (APC-Cy7; Biolegend) and CD14 (PE-Cy7; Biolegend) or corresponding isotype control antibodies for 15 min at room temperature. Cells were washed, then cells were fixed and RBCs lysed using the Immuno-Lyse and Fixative kit (Beckman Coulter) according to manufacturer's instructions. These were then washed and fluorescence in samples and controls (isotype, unstained, and single stains) was evaluated on a BD LSRFortessa flow cytometer.

Lipid mediator receptor expression on peripheral blood leukocytes

Unconjugated antibodies against ALX/FPR2 (Novus), LGR6 (Stratech), GPR101 (Antibodies-online), GPR37 (Invitrogen), GPR32 (Genetex), and GPR18 (Biobyrt) were conjugated with the fluorophores described below using Lightning-Link conjugation kits (Abcam) or APEX Antibody labelling kits (Invitrogen) according to manufacturer's instructions. Whole blood was transferred to 15ml tubes (50µl/experimental condition). Samples were centrifuged 5 min at 2000 x *g* and cell pellets were washed with Ca^{2+}/Mg^{2+}-free PBS containing 0.02% (w/v) bovine serum albumin. Samples were then stained with fluorophore-conjugated antibodies against human CD16 (APC-Cy7; Biolegend), CD14 (PE-Cy7; Biolegend), ALX/FPR2 (AF 594), LGR6 (PerCP-Cy5.5), GPR101 (AF 488), GPR37 (AF 700), GPR32 (Pacific Blue), GPR18 (AF 647), and ChemR23 (PE; R&D Systems) or corresponding isotype control antibodies for 15 min at room temperature. Cells were washed then fixed and RBCs lysed using Whole Blood Immuno-Lyse and Fixative kit (Beckman Coulter) according to manufacturer's instructions. These cells were then washed and fluorescence in samples and controls (isotype, unstained, and single stains) was evaluated on a BD LSRFortessa flow cytometer.

Lipid mediator biosynthetic enzyme expression in peripheral blood leukocytes

Unconjugated antibodies against ALOX12 (Abgent) and ALOX15B (Sigma) were conjugated with the fluorophores described below using Lightning-Link conjugation kits (Abcam) or APEX Antibody labelling kits (Invitrogen) according to manufacturer's instructions. Whole blood was transferred to 15ml tubes (50µl/experimental condition). Samples were centrifuged 5 min at 2000 x *g* and cell pellets were washed with Ca^{2+}/Mg^{2+}-free PBS containing 0.02% (w/v) bovine serum albumin. Samples were then stained with fluorophore-conjugated antibodies against human CD16 (APC-Cy7; Biolegend) and CD14 (PE-Cy7; Biolegend) or corresponding isotype control antibodies for 15 min at room temperature. Samples were washed, then RBCs were lysed and cells fixed and permeabilized using the Immuno-Lyse and Fixative kit (Beckman Coulter) according to manufacturer's instructions. Permeabilized cells were then further stained with fluorophore-conjugated antibodies against human COX-2 (AF 488; Abcam), ALOX5 (DyLight 405; Novus), ALOX12 (AF 594), ALOX15 (AF 647; Bioss Antibodies), and 15-LOX-2 (AF 700) or corresponding isotype control antibodies for 30 min at

4°C. Samples were then washed and fluorescence in samples and controls (isotype, unstained, and single stains) was evaluated on a BD LSRFortessa flow cytometer.

Neutrophil and monocyte assays

Bacterial particle phagocytosis by isolated neutrophils and monocytes

Streptococcus pneumoniae (NCTC 7465; Public Health England) was cultured in Brain Heart Infusion Broth for 16 hrs at 37°C and 250 rpm shaking. Bacterial cultures were pelleted by centrifugation at 5000 x g for 20 min, stained with 1µM pHrodo Red Succinimidyl ester in HBSS for 45 min at room temperature, washed with HBSS, fixed with 100% methanol, then washed again and resuspended in Ca^{2+}/Mg^{2+}-free PBS. pHrodo Red-conjugated *S. pneumoniae* particles, pHrodo Green-conjugated *S. aureus* particles (Invitrogen), and pHrodo Red-conjugated zymosan particles (Invitrogen) were opsonized by incubating in 20% (v/v) human serum (male A/B; Sigma) for 30 min at 37°C. PMN and monocytes were isolated from whole blood by Histopaque-1077 density centrifugation as described above. Neutrophils (5 x 10^6 cells/donor) or mononuclear cells (1 x 10^6 cells/donor) were transferred to 15ml tubes and nuclei were counter-stained in suspension with 1µg/ml Hoechst 33342 (Invitrogen) for 15 min at 37°C. Cells were washed, resuspended in X-VIVO15 medium (Lonza) containing 2mM L-glutamine (Sigma) and 1% Penicillin/Streptomycin solution (Sigma), and seeded in 96-well plates at 250.000 cells/well (PMNs) or 50.000 cells/well (PBMCs). Cells were allowed to adhere for 30 min at 37°C and then treated with 0.1nM or 1nM MCTR3, PCTR3, 17R-RvD3, RvD2, or corresponding vehicle control (0.001-0.01% ethanol) in X-VIVO15 medium for 15 min at 37°C, followed by addition of 25µl opsonized pHrodo Red-conjugated *S. pneumoniae* (5µg/well), pHrodo Green-conjugated *S. aureus* (5µg/well), or pHrodo Red-conjugated zymosan (2.5µg/well). The increase in pHrodo Red/Green signal over time, indicative of bioparticle phagocytosis, was then quantified using a Celldiscoverer 7 high-content imaging system (Zeiss) over a 2 hr period.

Adhesion molecule expression on isolated neutrophils and monocytes

Neutrophils and monocytes isolated from whole blood by Histopaque-1077 density centrifugation were transferred to 15ml tubes (250.000 cells/condition), blocked with 1mg/ml human IgG (Sigma), and stained with fluorophore-conjugated antibodies against CD16 (APC-Cy7; Biolegend), CD14 (PE-Cy7; Biolegend), CD32 (PE-Cy7; Biolegend), CD11b (BV711; Biolegend), CD49d (PE/Dazzle 594; Biolegend), CD54 (AF488; Biolegend), CD162 (Pacific Blue; Biolegend), and CD142 (APC; Biolegend) or corresponding isotype control antibodies for 15 min at room temperature. Cells were then washed and fixed using the Intracellular Fixation & Permeabilization Buffer Set (eBioscience) according to manufacturer's instructions. Samples were then washed and fluorescent stainings in samples and controls (isotype, unstained, and single stains) were evaluated on a BD LSRFortessa flow cytometer.

Monocyte-derived macrophage assays

Bacterial particle phagocytosis by cultured monocyte-derived macrophage

PBMCs isolated from whole blood by Histopaque-1077 density centrifugation were seeded at 100.000 cells/well in 96-well plates. Cells were allowed to adhere for 45 min at 37°C. Adherent cells were then washed with Ca^{2+}/Mg^{2+}-free PBS and incubated at 37°C and 5% CO_2 in RPMI-1640 medium (Sigma) containing 10% human serum (male A/B; Sigma), 20 ng/mL granulocyte-macrophage colony-stimulating factor (Peprotech), and 1% Penicillin/Streptomycin solution (Sigma) for 7 days (with medium being refreshed after 3 days) to differentiate cells to monocyte-derived macrophages (MoDMs). After 7 days, macrophages were washed and their nuclei were counter-stained with 1µg/ml Hoechst 33342 (Invitrogen) for 15 min at 37°C. pHrodo Red-conjugated *S. pneumoniae* particles, pHrodo Green-conjugated *S. aureus* particles (Invitrogen), and pHrodo Red-conjugated zymosan particles (Invitrogen) were opsonized by incubating in 20% (v/v) human serum (male A/B; Sigma) for 30 min at 37°C.

Macrophages were then treated with 0.1nM or 1nM MCTR3, PCTR3, 17R-RvD3, RvD2, or corresponding vehicle control (0.001-0.01% ethanol) for 15 min at 37°C, followed by addition of 25µl opsonized pHrodo Red-conjugated *S. pneumoniae* (5µg/well), pHrodo Green-conjugated *S. aureus* (5µg/well), or pHrodo Red-conjugated zymosan (2.5µg/well). The increase in pHrodo Red/Green signal

over time, indicative of bioparticle phagocytosis, was then quantified using a Celldiscoverer 7 high-content imaging system (Zeiss) over a 2 h period.

Apoptotic cell efferocytosis by cultured monocyte-derived macrophages
PBMCs isolated from whole blood by Histopaque-1077 density centrifugation were seeded at 100.000 cells/well in 96-well plates and allowed to adhere for 45 min at 37°C. Adherent cells were washed with Ca^{2+}/Mg^{2+}-free PBS and incubated at 37°C and 5% CO_2 in RPMI-1640 medium (Sigma) containing 10% human serum (male A/B; Sigma), 20 ng/mL GM-CSF (Peprotech), and 1% Penicillin/Streptomycin solution (Sigma) for 7 days (with medium being refreshed after 3 days) to differentiate cells to monocyte-derived macrophages. After 7 days, MoDMs were washed and their nuclei were counter-stained with 1µg/ml Hoechst 33342 (Invitrogen) for 15 min at 37°C. Apoptotic cells for efferocytosis were generated by irradiating 1.5 x 10^6 human promyelocytic HL-60 cells in 35-mm plates with 254 nm UV-C light for 15 min, followed by incubation at 37°C and 5% CO_2 for 2 hrs. Induction of apoptosis was verified using the APC Annexin V Apoptosis Detection Kit with PI (Biolegend) and flow cytometry, where Annexin V-positive and Annexin V/PI-double positive cells were considered apoptotic. Apoptotic HL-60 cells were washed, labelled with 1 µM pHrodo Red Succinimidyl Ester (Invitrogen) in Ca^{2+}/Mg^{2+}-free PBS for 30 min at room temperature, and resuspended in Ca^{2+}/Mg^{2+}-free PBS. Monocyte-derived macrophage were treated with 0.1nM or 1nM MCTR3, PCTR3, 17R-RvD3, RvD2, or corresponding vehicle control (0.001-0.01% ethanol) for 15 min at 37°C and 5% CO_2, followed by addition of pHrodo Red-labelled apoptotic cells in a 3:1 ratio (apoptotic HL-60s:monocyte-derived macrophage). The increase in pHrodo Red signal over time, indicative of apoptotic cell efferocytosis, was quantified using a Celldiscoverer 7 high-content imaging system (Zeiss) over a 2 hr period.

Cytokine expression in cultured monocyte-derived macrophages
An unconjugated antibody against human IFN-α subtypes 1/13, 2, 4, 5, 6, 7, 8, 10, 14, 16, and 17 (pan-IFN-α; MAbTech) was conjugated with AF 488 using a Lightning-Link conjugation kit (Abcam) according to manufacturer's instructions. PBMCs isolated from whole blood by Histopaque-1077 density centrifugation were seeded at 400.000 cells/well in low-attachment 24-well plates. Cells were allowed to adhere for 45 min at 37°C. Adherent cells were then washed with Ca^{2+}/Mg^{2+}-free PBS and incubated at 37°C and 5% CO_2 in RPMI-1640 medium (Sigma) containing 10% human serum (male A/B; Sigma), 20 ng/mL GM-CSF (Peprotech), 1% Penicillin/Streptomycin solution (Sigma), and 10nM MCTR3, PCTR3, 17R-RvD3, RvD2, or corresponding vehicle control (0.01% ethanol) for 7 days (with medium being refreshed after 3 days) to differentiate cells to monocyte-derived macrophages (MoDMs). After 7 days, macrophages were washed and stimulated with 1µg/ml recombinant human S100A8/A9 dimer (Biolegend) for 24 hrs to induce cytokine expression. For the final 18 hrs of S100A8/A9 stimulation, 2µg/ml Brefeldin A (Biolegend) was added to induce intracellular accumulation of cytokines. Unstimulated control wells were only treated with 2µg/ml Brefeldin A. Cells were then washed, detached by incubating in Ca^{2+}/Mg^{2+}-free PBS containing 5mM EDTA for 15 min at 37°C, blocked with 1mg/ml human IgG (Sigma), and stained with fluorophore-conjugated antibody against CD14 (APC-Cy7; Biolegend) or APC-Cy7 isotype control antibody for 15 min at room temperature. Cells were then washed, fixed and permeabilized using the Intracellular Fixation & Permeabilization Buffer Set (eBioscience) according to manufacturer's instructions, and further stained using fluorophore-conjugated antibodies against pan-IFN-α (AF 488), TNF-α (BV650; Biolegend), IL-1β (AF 647; Biolegend), IL-6 (PerCP/Cy5.5; Biolegend), and IL-10 (BV786; BD Biosciences) or corresponding isotype control antibodies for 30 min at 4°C. Samples were then washed and fluorescent staining in samples and controls (isotype, unstained, and single stains) were evaluated on a BD LSRFortessa flow cytometer.

Macrophage phenotype marker expression in cultured monocyte-derived macrophages
PBMCs isolated from whole blood by Histopaque-1077 density centrifugation were seeded at 400.000 cells/well in low-attachment 24-well plates. Cells were allowed to adhere for 45 min at 37°C. Adherent cells were then washed with Ca^{2+}/Mg^{2+}-free PBS and incubated at 37°C and 5% CO_2 in RPMI-1640 medium (Sigma) containing 10% human serum (male A/B; Sigma), 20 ng/mL GM-CSF (Peprotech), 1% Penicillin/Streptomycin solution (Sigma), and 10nM MCTR3, PCTR3, 17R-RvD3, RvD2, or

corresponding vehicle control (0.01% ethanol) for 7 days (with medium being refreshed after 3 days) to differentiate cells to monocyte-derived macrophages. After 7 days, macrophages were washed, detached by incubating in Ca^{2+}/Mg^{2+}-free PBS containing 5mM EDTA for 15 min at 37°C, blocked with 1mg/ml human IgG (Sigma), and stained with fluorophore-conjugated antibodies against CD14 (APC-Cy7; Biolegend), CD32 (PE-Cy7; Biolegend), CD64 (PE-Cy5; Biolegend); CD68 (AF 488; Biolegend), CD80 (BV650; Biolegend), CD206 (PerCP-Cy5.5; Biolegend), CD163 (PE-CF594; BD Biosciences), and CD142 (APC; Biolegend) or corresponding isotype control antibodies for 15 min at room temperature. Cells were then washed, fixed and permeabilized using the Intracellular Fixation & Permeabilization Buffer Set (eBioscience) according to manufacturer's instructions, and further stained using fluorophore-conjugated antibodies against Arg1 (PE; R&D Systems) and MerTK (BV421; Biolegend) or corresponding isotype control antibodies for 30 min at 4°C. Samples were then washed and fluorescence in samples and controls (isotype, unstained, and single stains) was evaluated on a BD LSRFortessa flow cytometer.

Data analysis and statistical testing

Flow cytometry data was analyzed using FlowJo v10.4. For Partial Least Square Discriminant analyses (PLS-DA), missing values were replaced by 1/5th of the minimum value of each variable across the samples, and features with a constant or single value across samples were removed. Data was then auto-scaled, 2D plots of Component 1 and 2 from each PLS-DA with their 95% confidence intervals (CI) were constructed, Variable Importance in Projection (VIP) scores were calculated, and internal validation by leave-one-out cross-validation and calculation of a coefficient of determination (R^2) for each model was performed using MetaboAnalyst[54]. Mahalanobis distances between group centers and Hotelling's T-squared test p-values were calculated for Component 1 and 2 scores of the PLS-DA models using *pca-utils*[55]. For correlation analyses of plasma LM versus either phagocyte activation markers or bacterial phagocytosis, tables were constructed in which plasma LM quantities (in pg/ml) and phagocyte activation marker expression or bacterial phagocytosis (in MFI) were reported for each patient sample. Pearson correlation coefficients between plasma LM levels and activation marker MFIs were calculated using MetaboAnalyst, transformed into absolute values, plotted in Circos format using Circos Table Viewer[56] and coloured according to correlation direction (i.e. positive or negative). All other statistical tests were performed using GraphPad Prism 9 and Microsoft Excel. Results are presented as mean ± SEM for bar graphs and median plus interquartile range for violin plots. Statistical significance was determined as described in the figure legends. Briefly, normal distribution of data was tested using the Shapiro-Wilk normality test. For non-normal distributed data or comparisons where group sizes were too small to reliably determine normality ($n<6$), the Mann-Whitney U test was used for unpaired samples and the Wilcoxon signed rank test was used for paired samples. Where indicated in the figure legends, correction for multiple testing was performed using Dunn's post-hoc test correction for multiple testing was performed using Dunn's post-hoc test. Across-test corrections for multiple testing were not performed.

SUPPLEMENTAL TABLES

Supplemental Table I: Clinical characteristics of healthy volunteers, COVID-19 patients, and Post-COVID-19 volunteers.

Characteristics	Healthy	COVID-19	Post-COVID-19
n	12	39	8
**Age**	33 (22-54)	55 (26-83)	33 (26-52)
**Male**	5 (41.7%)	26 (66.7%)	3 (37.5%)
**Ethnicity**			
Black British	1 (8.3%)	4 (10.3%)	0
Indian British or Other Asian	2 (16.7%)	16 (41.0%)	3 (37.5%)
White British or Other White	9 (75.0%)	17 (43.6%)	5 (62.5%)
**Days since symptom onset**	–	11 (5-37)	28 (21-49)
**Days since symptom resolution**	–	–	21 (12-25)
**Days since hospitalisation**	–	2 (0-13)	–
**WHO Ordinal Scale (at time of sample)**	0	3-5	0
**Comorbidities**			
Past smoking history	0	9 (23.1%)	0
Obesity (BMI > 30)	0	15 (38.5%)	0
Type 2 diabetes mellitus	0	10 (25.6%)	0
Hypercholesterolaemia	0	12 (30.8%)	0
Hypertension	0	13 (33.3%)	0
*Heart disease **	0	6 (15.4%)	0
Asthma	2 (16.6%)	8 (20.5%)	0
Chronic obstructive pulmonary disease	0	3 (7.7%)	0
Chronic kidney disease	0	1 (2.6%)	0
None of the above	10 (83.3%)	3 (7.7%)	8 (100%)
**Medications ****			
Dexamethasone	–	28 (71.8%)	0
Remdesivir	–	5 (12.8%)	0
Aspirin	–	3 (7.7%)	0
Colchicine	–	3 (7.7%)	0
Hydroxychloroquine	–	1 (2.6%)	0
Tocilizumab	–	1 (2.6%)	0
**Oxygen therapy**			
Room air	–	5 (12.8%)	–
Nasal cannula/Venturi mask	–	20 (51.3%)	–
High-flow nasal oxygen	–	14 (35.9%)	–

** Includes ischemic heart disease, atrial fibrillation, left ventricular failure, and valvular heart disease.*
*** Specific COVID-19 medications given in addition to the patient's usual medications, antibiotics and thromboembolic prophylaxis as indicated.*

Data are shown as number (percentage). Age is reported as median years (range). Days since symptom onset/symptom resolution/hospitalisation are reported as median days (range) at time of sample collection. Not all data were collected for Healthy and Post COVID-19 individuals.

Supplemental Table II: Plasma lipid mediator profiles in healthy volunteers, COVID-19 patients, and Post-COVID-19 volunteers.

				pg/mL of plasma					
				Healthy		COVID-19		Post-COVID-19	
	Q1	Q3	IS used	Mean±SEM	No of samples identified in	Mean±SEM	No of samples identified in	Mean±SEM	No of samples identified in
**DHA Bioactive Metabolome**									
RvD1	375	215	d_5-17R-RvD1	0.57±0.05	2	0.65±0.17	3	2.07±1.06	2
RvD2	375	141	d_5-RvD2	0.49±0.00	1	1.41±0.71	2	0.64±0.16	3
RvD3	375	147	d_5-RvD3	0.20±0.12	2	0.22±0.09	4	-	-
RvD4	375	101	d_5-17R-RvD1	0.42±0.12	3	0.49±0.20	6	0.49±0.00	1
RvD5	359	199	d_5-MaR1	0.12±0.02	6	0.35±0.08	10	-	-
RvD6	359	101	d_5-MaR1	-	-	1.06±0.22	10	0.20±0.00	1
17R-RvD1	375	215	d_5-17R-RvD1	0.64±0.00	1	1.18±0.44	3	-	-
17R-RvD3	375	147	d_5-RvD3	-	-	0.18±0.06	6	0.04±0.00	1
PD1	359	153	d_5-MaR1	0.08±0.01	3	0.58±0.11	11	0.07±0.02	2
17R-PD1	359	153	d_5-MaR1	-	-	0.05±0.00	1	-	-
10S,17S-diHDHA	359	153	d_5-MaR1	0.08±0.00	1	0.54±0.11	2	0.06±0.00	2
PCTR1	650	231	d_5-LTC$_4$	-	-	2.67±1.54	2	-	-
PCTR2	521	231	d_5-LTD$_4$	-	-	2.10±0.47	5	-	-
PCTR3	464	231	d_5-LTE$_4$	-	-	108.54±43.29	10	24.48±0.00	1
MaR1	359	221	d_5-MaR1	2.26±0.50	4	7.87±2.91	2	1.28±0.54	2
MaR2	359	221	d_5-MaR2	-	-	-	-	-	-
7S,14S-diHDHA	359	221	d_5-MaR1	1.09±0.18	4	5.37±1.40	14	0.66±0.00	1
4S,14S-diHDHA	359	101	d_5-MaR2	0.47±0.00	1	0.85±0.32	4	0.11±0.00	1
MCTR1	650	191	d_5-LTC$_4$	-	-	2.34±0.00	1	-	-
MCTR2	521	191	d_5-LTD$_4$	2.12±0.46	2	1.94±0.60	5	-	-
MCTR3	464	191	d_5-LTE$_4$	-	-	42.9±18.7	14	12.86±0.00	1
**n-3 DPA Bioactive Metabolome**									
RvT1	377	193	d_5-RvD2	2.24±0.65	7	2.48±0.38	20	1.91±0.33	6
RvT2	377	197	d_5-17R-RvD1	0.43±0.00	1	0.71±0.09	3	2.35±1.62	2
RvT3	377	173	d_5-17R-RvD1	0.33±0.00	1	0.32±0.04	4	-	-
RvT4	361	211	d_5-MaR1	0.14±0.04	2	0.94±0.20	5	-	-
RvD1$_{n-3DPA}$	377	215	d_5-17R-RvD1	0.98±0.17	4	0.99±0.48	2	2.14±0.00	1
RvD2$_{n-3DPA}$	377	233	d_5-RvD2	5.22±2.49	3	13.04±5.37	4	9.44±5.67	3
RvD5$_{n-3DPA}$	361	199	d_5-MaR1	0.19±0.07	3	1.69±0.49	12	0.13±0.00	1
PD1$_{n-3\ DPA}$	361	183	d_5-MaR1	0.04±0.00	1	0.26±0.07	9	0.05±0.00	1
PD2$_{n-3\ DPA}$	361	233	d_5-MaR2	-	-	-	-	-	-
10S,17S-diHDPA	361	183	d_5-MaR1	0.14±0.05	4	0.33±0.18	4	0.19±0.07	3
MaR1$_{n-3\ DPA}$	361	223	d_5-MaR1	0.35±0.12	2	1.56±0.64	3	0.36±0.08	2
MaR2$_{n-3\ DPA}$	361	223	d_5-MaR2	0.74±0.25	11	0.77±0.15	11	1.39±0.65	5
7S,14S-diHDPA	361	223	d_5-MaR1	0.59±0.39	2	2.31±0.00	1	-	-
**EPA Bioactive Metabolome**									
RvE1	349	161	d_4-RvE1	-	-	0.88±0.00	1	-	-
RvE2	333	159	d_4-LTB$_4$	-	-	4.76±0.00	1	-	-
RvE3	333	275	d_4-LTB$_4$	2.20±0.20	2	9.52±2.28	6	6.21±0.00	1
RvE4	333	115	d_4-LTB$_4$	0.80±0.38	11	6.72±1.60	31	0.96±0.28	6
**AA Bioactive Metabolome**									
LXA$_4$	351	115	d_5-LXA$_4$	-	-	0.99±0.23	12	-	-
LXB$_4$	351	221	d_5-LXA$_4$	1.13±0.00	1	1.72±0.72	3	1.55±0.75	2
5S,15S-diHETE	335	235	d_4-LTB$_4$	3.95±2.41	8	7.58±1.87	20	2.04±0.38	5
15-epi-LXA$_4$	351	115	d_5-LXA$_4$	0.78±0.08	8	1.51±0.33	10	0.37±0.01	2
15-epi-LXB$_4$	351	221	d_5-LXA$_4$	55.86±44.25	6	19.58±13.24	10	13.89±6.76	4
LTB$_4$	335	195	d_4-LTB$_4$	0.69±0.49	11	1.94±0.45	31	1.05±0.62	7
5S,12S-diHETE	335	195	d_4-LTB$_4$	0.25±0.10	4	0.98±0.13	24	0.72±0.36	4
6-trans-LTB$_4$	335	195	d_4-LTB$_4$	0.17±0.04	7	0.50±0.10	16	0.20±0.08	3
6-trans-12-epi LTB$_4$	335	195	d_4-LTB$_4$	0.22±0.07	9	1.26±0.36	17	0.39±0.17	5
LTC$_4$	626	189	d_5-LTC$_4$	4.64±1.78	3	6.65±0.00	1	6.54±4.58	3
LTD$_4$	497	189	d_5-LTD$_4$	3.44±0.00	1	0.81±0.18	6	3.11±2.62	2
LTE$_4$	440	189	d_5-LTE$_4$	2.46±1.69	4	2.04±0.27	29	4.36±1.36	4
PGD$_2$	351	189	d_4-PGE$_2$	6.69±1.67	7	16.94±6.45	31	16.84±4.85	5
PGE$_2$	351	189	d_4-PGE$_2$	1.43±0.64	7	4.68±3.06	25	4.07±1.68	7
PGF$_{2\alpha}$	353	193	d_4-PGE$_2$	4.53±0.00	1	7.46±4.42	22	10.19±0.95	5
TXB$_2$	369	169	d_4-PGE$_2$	33.59±14.51	7	187.37±89.65	20	183.47±96.29	6

Results are reported as pg mediator/mL of plasma for all samples in which that mediator was identified. n=12 for healthy volunteers, n=38 for COVID-19 patients, and n=8 for Post-COVID-19 volunteers.

Supplemental Table III: Clinical characteristics of COVID-19 patients separated by disease severity.

Characteristics	COVID-19		
	Mild (WHO 3)	*Mild (WHO 4)*	*Severe (WHO 5)*
n	5	20	14
**Age**	63 (43-79)	55 (26-83)	58 (27-75)
**Male**	2 (40.0%)	12 (48.0%)	12 (85.7%)
**Days since symptom onset**	15 (12-37)	10 (5-23)	11 (7-30)
**Days since hospitalisation**	7 (2-13)	2 (0-12)	3.5 (0-9)
**Discharge status: alive**	5 (100%)	19 (95.0%)	11 (78.6%)
**WHO Ordinal Scale (at time of sample)**	3	4	5
**Disease trajectory (at time of sample)**			
Improving	3 (60.0%)	6 (30.0%)	2 (14.3%)
Stable	2 (40.0%)	11 (55.0%)	4 (28.6%)
Deteriorating	0	3 (15.0%)	8 (57.1%)
**Comorbidities**			
Past smoking history	1 (20.0%)	5 (25.0%)	3 (21.4%)
Obesity (BMI > 30)	3 (60.0%)	9 (45.0%)	3 (21.4%)
Type 2 diabetes mellitus	2 (40.0%)	3 (15.0%)	5 (35.7%)
Hypercholesterolaemia	2 (40.0%)	5 (25.0%)	5 (35.7%)
Hypertension	3 (60.0%)	4 (20.0%)	6 (42.9%)
*Heart disease **	1 (20.0%)	2 (10.0%)	3 (21.4%)
Asthma	1 (20.0%)	3 (15.0%)	4 (28.6%)
Chronic obstructive pulmonary disease	1 (20.0%)	2 (10.0%)	0
Chronic kidney disease	1 (20.0%)	0	0
None of the above	0	2 (10.0%)	1 (7.1%)
**Medications ****			
Dexamethasone	2 (40.0%)	16 (80.0%)	10 (71.4%)
Remdesivir	0	2 (10.0%)	3 (21.4%)
Aspirin	0	2 (10.0%)	1 (7.1%)
Colchicine	0	0	3 (21.4%)
Hydroxychloroquine	0	0	1 (7.1%)
Tocilizumab	0	0	1 (7.1%)
**Oxygen therapy**			
Room air	5 (100%)	0	0
Nasal cannula/Venturi mask	0	20 (100%)	0
High-flow nasal oxygen	0	0	14 (100%)

** Includes ischemic heart disease, atrial fibrillation, left ventricular failure, and valvular heart disease.*
*** Specific COVID-19 medications given in addition to the patient's usual medications, antibiotics and thromboembolic prophylaxis as indicated.*

Data are shown as number (percentage). Age is reported as median years (range). Days since symptom onset/hospitalisation are reported as median days (range) at time of sample collection.

Supplemental Table IV: Plasma lipid mediator profiles in COVID-19 patients separated by disease severity.

				pg/mL of plasma			
				WHO 3-4		WHO 5	
	Q1	Q3	IS used	Mean±SEM	No of samples identified in	Mean±SEM	No of samples identified in
**DHA Bioactive Metabolome**							
RvD1	375	215	d_5-17R-RvD1	0.99±0.00	1	0.49±0.03	2
RvD2	375	141	d_5-RvD2	1.41±0.71	2	-	-
RvD3	375	147	d_5-RvD3	0.17±0.13	2	0.26±0.16	2
RvD4	375	101	d_5-17R-RvD1	0.49±0.20	6	-	-
RvD5	359	199	d_5-MaR1	0.37±0.13	6	0.31±0.09	4
RvD6	359	101	d_5-MaR1	1.00±0.26	6	1.14±0.43	4
17R-RvD1	375	215	d_5-17R-RvD1	1.18±0.44	3	-	-
17R-RvD3	375	147	d_5-RvD3	0.15±0.04	4	0.24±0.19	2
PD1	359	153	d_5-MaR1	0.50±0.14	8	0.78±0.13	3
17R-PD1	359	153	d_5-MaR1	0.05±0.00	1	-	-
10S,17S-diHDHA	359	153	d_5-MaR1	0.65±0.00	1	0.44±0.00	1
PCTR1	650	231	d_5-LTC_4	1.13±0.00	1	4.21±0.00	1
PCTR2	521	231	d_5-LTD_4	2.33±0.81	3	1.76±0.17	2
PCTR3	464	231	d_5-LTE_4	117.44±47.37	9	28.4±0.00	1
MaR1	359	221	d_5-MaR1	4.96±0.00	1	10.78±0.00	1
MaR2	359	221	d_5-MaR2	-	-	-	-
7S,14S-diHDHA	359	221	d_5-MaR1	5.41±1.78	10	5.28±2.43	4
4S,14S-diHDHA	359	101	d_5-MaR2	0.52±0.08	2	1.19±0.63	2
MCTR1	650	191	d_5-LTC_4	-	-	2.34±0.00	1
MCTR2	521	191	d_5-LTD_4	1.91±0.77	4	2.08±0.00	1
MCTR3	464	191	d_5-LTE_4	51.82±23.23	11	10.19±6.09	3
**n-3 DPA Bioactive Metabolome**							
RvT1	377	193	d_5-RvD2	2.64±0.44	17	1.56±0.38	3
RvT2	377	197	d_5-17R-RvD1	0.80±0.02	2	0.54±0.00	1
RvT3	377	173	d_5-17R-RvD1	0.30±0.05	3	0.38±0.00	1
RvT4	361	211	d_5-MaR1	0.94±0.20	5	-	-
$RvD1_{n-3DPA}$	377	215	d_5-17R-RvD1	0.99±0.48	2	-	-
$RvD2_{n-3DPA}$	377	233	d_5-RvD2	13.04±5.37	4	-	-
$RvD5_{n-3DPA}$	361	199	d_5-MaR1	1.10±0.40	7	2.5±1.00	5
$PD1_{n-3\ DPA}$	361	183	d_5-MaR1	0.16±0.06	5	0.40±0.12	4
$PD2_{n-3\ DPA}$	361	233	d_5-MaR2	-	-	-	-
10S,17S-diHDPA	361	183	d_5-MaR1	0.16±0.08	3	0.84±0.00	1
$MaR1_{n-3\ DPA}$	361	223	d_5-MaR1	2.16±0.00	1	1.27±0.99	2
$MaR2_{n-3\ DPA}$	361	223	d_5-MaR2	0.72±0.24	6	0.83±0.19	5
7S,14S-diHDPA	361	223	d_5-MaR1	-	-	2.31±0.00	1
**EPA Bioactive Metabolome**							
RvE1	349	161	d_4-RvE1	0.88±0.00	1	-	-
RvE2	333	159	d_4-LTB_4	4.76±0.00	1	-	-
RvE3	333	275	d_4-LTB_4	9.04±2.73	5	11.91±0.00	1
RvE4	333	115	d_4-LTB_4	7.79±2.06	22	4.10±2.13	9
**AA Bioactive Metabolome**							
LXA_4	351	115	d_5-LXA_4	0.90±0.24	6	1.09±0.42	6
LXB_4	351	221	d_5-LXA_4	-	-	1.72±0.72	3
5S,15S-diHETE	335	235	d_4-LTB_4	7.71±2.18	14	7.30±3.89	6
15-epi-LXA_4	351	115	d_5-LXA_4	1.83±0.46	6	1.03±0.39	4
15-epi-LXB_4	351	221	d_5-LXA_4	26.73±18.64	7	2.91±0.42	3
LTB_4	335	195	d_4-LTB_4	1.39±0.33	19	2.81±1.01	12
5S,12S-diHETE	335	195	d_4-LTB_4	0.84±0.14	16	1.25±0.28	8
6-trans-LTB_4	335	195	d_4-LTB_4	0.49±0.12	13	0.52±0.24	3
6-trans-12-epi LTB_4	335	195	d_4-LTB_4	0.79±0.17	11	2.12±0.93	6
LTC_4	626	189	d_5-LTC_4	6.65±0.00	1	-	-
LTD_4	497	189	d_5-LTD_4	0.70±0.18	4	1.03±0.49	2
LTE_4	440	189	d_5-LTE_4	1.48±0.19	16	2.73±0.49	13
PGD_2	351	189	d_4-PGE_2	20.77±9.82	20	9.98±3.32	11
PGE_2	351	189	d_4-PGE_2	6.70±4.76	16	1.09±0.45	9
$PGF_{2\alpha}$	353	193	d_4-PGE_2	10.89±7.35	13	2.51±1.67	9
TXB_2	369	169	d_4-PGE_2	241.42±133.7	13	87±61.15	7

Results are reported as pg mediator/mL of plasma for all samples in which that mediator was identified. n=25 for COVID-19 patients at WHO scale 3-4, n=13 for COVID-19 patients at WHO scale 5.

Supplemental Table V: Clinical characteristics of COVID-19 patients separated by dexamethasone treatment.

Characteristics	COVID-19	
	No Dexamethasone	*Dexamethasone (6mg/daily)*
n	11	28
**Age**	60 (39-69)	55 (26-83)
**Male**	8 (72.7%)	18 (64.3%)
**Days since symptom onset**	12 (6-37)	10 (5-23)
**Days since hospitalisation**	3 (0-9)	2 (0-13)
**Discharge status: alive**	1 (9.1%)	3 (10.7%)
**WHO Ordinal Scale (at time of sample)**		
Scale 3	3 (27.2%)	2 (7.1%)
Scale 4	4 (36.4%)	16 (57.1%)
Scale 5	4 (36.4%)	10 (35.7%)
**Comorbidities**		
Past smoking history	2 (18.2%)	7 (25.0%)
Obesity (BMI > 30)	5 (45.5%)	10 (35.7%)
Type 2 diabetes mellitus	3 (27.3%)	7 (25.0%)
Hypercholesterolaemia	5 (45.5%)	7 (25.0%)
Hypertension	6 (54.5%)	7 (25.0%)
*Heart disease **	0	6 (21.4%)
Asthma	3 (27.3%)	5 (17.9%)
Chronic obstructive pulmonary disease	1 (9.1%)	2 (7.1%)
Chronic kidney disease	0	1 (3.6%)
None of the above	1 (9.1%)	2 (7.1%)
**Medications ****		
Dexamethasone	0	28 (100%)
Remdesivir	0	5 (17.9%)
Aspirin	0	3 (10.7%)
Colchicine	0	3 (10.7%)
Hydroxychloroquine	1 (9.1%)	0
Tocilizumab	0	1 (3.6%)
**Oxygen therapy**		
Room air	3 (27.3%)	2 (7.1%)
Nasal cannula/Venturi mask	4 (36.4%)	16 (57.4%)
High-flow nasal oxygen	4 (36.4%)	10 (35.7%)

** Includes ischemic heart disease, atrial fibrillation, left ventricular failure, and valvular heart disease.*
*** Specific COVID-19 medications given in addition to the patient's usual medications, antibiotics and thromboembolic prophylaxis as indicated.*

Data are shown as number (percentage). Age is reported as median years (range). Days since symptom onset/hospitalisation are reported as median days (range) at time of sample collection.

Supplemental Table VI: Plasma lipid mediator profiles in COVID-19 patients separated by dexamethasone treatment.

				pg/mL of plasma			
				COVID-19		COVID-19 + Dex	
	Q1	Q3	IS used	Mean±SEM	No of samples identified in	Mean±SEM	No of samples identified in
**DHA Bioactive Metabolome**							
RvD1	375	215	d_5-17R-RvD1	0.49±0.03	2	0.99±0.00	1
RvD2	375	141	d_5-RvD2	0.70±0.00	1	2.12±0.00	1
RvD3	375	147	d_5-RvD3	0.05±0.00	1	0.28±0.09	3
RvD4	375	101	d_5-17R-RvD1	0.16±0.00	1	0.56±0.23	5
RvD5	359	199	d_5-MaR1	0.12±0.02	5	0.57±0.08	5
RvD6	359	101	d_5-MaR1	0.29±0.10	2	1.25±0.22	8
17R-RvD1	375	215	d_5-17R-RvD1	1.91±0.00	1	0.82±0.42	2
17R-RvD3	375	147	d_5-RvD3	0.04±0.00	2	0.25±0.06	4
PD1	359	153	d_5-MaR1	0.22±0.08	2	0.65±0.12	9
17R-PD1	359	153	d_5-MaR1	0.05±0.00	1	-	-
10S,17S-diHDHA	359	153	d_5-MaR1	-	-	0.54±0.11	2
PCTR1	650	231	d_5-LTC_4	-	-	2.67±1.54	2
PCTR2	521	231	d_5-LTD_4	-	-	2.10±0.47	5
PCTR3	464	231	d_5-LTE_4	251.81±51.08	4	13.02±4.19	6
MaR1	359	221	d_5-MaR1	-	-	7.87±2.91	2
MaR2	359	221	d_5-MaR2	-	-	-	-
7S,14S-diHDHA	359	221	d_5-MaR1	1.65±0.26	7	9.09±1.94	7
4S,14S-diHDHA	359	101	d_5-MaR2	-	-	0.85±0.32	4
MCTR1	650	191	d_5-LTC_4	-	-	2.34±0.00	1
MCTR2	521	191	d_5-LTD_4	3.08±0.00	1	1.66±0.68	4
MCTR3	464	191	d_5-LTE_4	128.55±42.90	4	8.64±2.34	10
**n-3 DPA Bioactive Metabolome**							
RvT1	377	193	d_5-RvD2	2.36±0.74	8	2.56±0.43	12
RvT2	377	197	d_5-17R-RvD1	-	-	0.71±0.09	3
RvT3	377	173	d_5-17R-RvD1	0.32±0.04	4	-	-
RvT4	361	211	d_5-MaR1	0.29±0.00	1	1.11±0.14	4
$RvD1_{n-3DPA}$	377	215	d_5-17R-RvD1	0.99±0.48	2	-	-
$RvD2_{n-3DPA}$	377	233	d_5-RvD2	5.77±0.51	2	20.32±8.19	2
$RvD5_{n-3DPA}$	361	199	d_5-MaR1	0.50±0.26	5	2.53±0.67	7
$PD1_{n-3\ DPA}$	361	183	d_5-MaR1	0.06±0.01	4	0.42±0.07	5
$PD2_{n-3\ DPA}$	361	233	d_5-MaR2	-	-	-	-
10S,17S-diHDPA	361	183	d_5-MaR1	0.08±0.01	2	0.58±0.26	2
$MaR1_{n-3\ DPA}$	361	223	d_5-MaR1	0.28±0.00	1	2.20±0.05	2
$MaR2_{n-3\ DPA}$	361	223	d_5-MaR2	-	-	0.77±0.15	11
7S,14S-diHDPA	361	223	d_5-MaR1	-	-	2.31±0.00	1
**EPA Bioactive Metabolome**							
RvE1	349	161	d_4-RvE1	-	-	0.88±0.00	1
RvE2	333	159	d_4-LTB_4	-	-	4.76±0.00	1
RvE3	333	275	d_4-LTB_4	3.42±0.92	2	12.57±1.88	4
RvE4	333	115	d_4-LTB_4	0.54±0.10	10	9.66±2.07	21
**AA Bioactive Metabolome**							
LXA_4	351	115	d_5-LXA_4	0.52±0.00	1	1.04±0.25	11
LXB_4	351	221	d_5-LXA_4	-	-	1.72±0.72	3
5S,15S-diHETE	335	235	d_4-LTB_4	1.60±0.24	8	11.57±2.53	12
15-epi-LXA_4	351	115	d_5-LXA_4	1.01±0.30	4	1.84±0.48	6
15-epi-LXB_4	351	221	d_5-LXA_4	2.43±0.77	2	23.87±16.39	8
LTB_4	335	195	d_4-LTB_4	0.54±0.11	9	2.51±0.59	22
5S,12S-diHETE	335	195	d_4-LTB_4	0.25±0.05	5	1.17±0.14	19
6-trans-LTB_4	335	195	d_4-LTB_4	0.11±0.03	5	0.67±0.11	11
6-trans-12-epi LTB_4	335	195	d_4-LTB_4	0.30±0.08	5	1.66±0.47	12
LTC_4	626	189	d_5-LTC_4	6.65±0.00	1	-	-
LTD_4	497	189	d_5-LTD_4	-	-	0.81±0.18	6
LTE_4	440	189	d_5-LTE_4	2.69±0.66	9	1.75±0.24	20
PGD_2	351	189	d_4-PGE_2	28.33±13.96	11	10.68±6.28	20
PGE_2	351	189	d_4-PGE_2	3.66±2.55	9	5.25±4.63	16
$PGF_{2\alpha}$	353	193	d_4-PGE_2	21.14±5.28	2	6.09±4.74	20
TXB_2	369	169	d_4-PGE_2	155.17±111.94	9	213.73±139.31	11

Results are reported as pg mediator/mL of plasma for all samples in which that mediator was identified. n=11 for COVID-19 patients, n=27 for COVID-19 + Dex patients.

Supplemental Table VII: Clinical characteristics of COVID-19 patients separated by disease trajectory.

Characteristics	COVID-19		
	Improving	*Stable*	*Deteriorating*
n	11	17	11
**Age**	53.5 (32-72)	57 (26-79)	55 (31-83)
**Male**	7 (63.6%)	9 (52.9%)	10 (90.9%)
**Days since symptom onset**	15 (7-37)	10 (6-15)	11 (5-25)
**Days since hospitalisation**	3 (1-17)	2 (0-13)	3 (0-14)
**Discharge status: alive**	11 (100%)	16 (94.1%)	8 (72.7%)
**WHO Ordinal Scale (at time of sample)**			
Scale 3	3 (27.3%)	2 (11.8%)	0
Scale 4	6 (54.5%)	11 (64.7%)	3 (27.3%)
Scale 5	2 (18.2%)	4 (23.5%)	8 (72.7%)
**Comorbidities**			
Past smoking history	1 (9.1%)	5 (29.4%)	3 (27.3%)
Obesity (BMI > 30)	8 (72.7%)	6 (35.3%)	3 (27.3%)
Type 2 diabetes mellitus	2 (18.2%)	2 (11.8%)	5 (45.4%)
Hypercholesterolaemia	5 (45.4%)	3 (17.6%)	4 (36.4%)
Hypertension	4 (36.4%)	5 (29.4%)	4 (36.4%)
*Heart disease **	1 (9.1%)	3 (17.6%)	2 (18.2%)
Asthma	1 (9.1%)	4 (23.5%)	3 (27.3%)
Chronic obstructive pulmonary disease	1 (9.1%)	2 (11.8%)	0
Chronic kidney disease	1 (9.1%)	0	0
None of the above	1 (9.1%)	1 (5.9%)	1 (9.1%)
**Medications ****			
Dexamethasone	7 (63.6%)	13 (76.5%)	8 (72.7%)
Remdesivir	0	4 (23.5%)	1 (9.1%)
Aspirin	3 (27.3%)	0	0
Colchicine	1 (9.1%)	1 (5.9%)	1 (9.1%)
Hydroxychloroquine	0	0	1 (9.1%)
Tocilizumab	0	0	1 (9.1%)
**Oxygen therapy**			
Room air	3 (27.3%)	2 (11.8%)	0
Nasal cannula/Venturi mask	6 (54.5%)	11 (64.7%)	3 (27.3%)
High-flow nasal oxygen	2 (18.2%)	4 (23.5%)	8 (72.7%)

** Includes ischemic heart disease, atrial fibrillation, left ventricular failure, and valvular heart disease.*

*** Specific COVID-19 medications given in addition to the patient's usual medications, antibiotics and thromboembolic prophylaxis as indicated.*

Data are shown as number (percentage). Age is reported as median years (range). Days since symptom onset/hospitalisation are reported as median days (range) at time of sample collection. The deteriorating group includes those patients that did not survive.

Supplemental Table VIII: Plasma lipid mediator profiles in COVID-19 patients separated by disease severity and trajectory.

				pg/mL of plasma							
				WHO 3-4; Improving/Stable		WHO 3-4; Deteriorating		WHO 5; Improving/Stable		WHO 5; Deteriorating	
	Q1	Q3	IS used	Mean±SEM	No of samples identified in	Mean±SEM	No of samples identified in	Mean±SEM	No of samples identified in	Mean±SEM	No of samples identified in
**DHA Bioactive Metabolome**											
RvD1	375	215	d_5-17R-RvD1	-	-	0.99±0.00	1	-	-	0.49±0.03	2
RvD2	375	141	d_5-RvD2	0.70±0.00	1	2.12±0.00	1	-	-	-	-
RvD3	375	147	d_5-RvD3	0.17±0.13	2	-	-	0.42±0.00	1	0.10±0.00	1
RvD4	375	101	d_5-17R-RvD1	0.56±0.23	5	0.18±0.00	1	-	-	-	-
RvD5	359	199	d_5-MaR1	0.43±0.15	5	0.09±0.00	1	0.26±0.13	2	0.36±0.17	2
RvD6	359	101	d_5-MaR1	1.08±0.30	5	0.58±0.00	1	1.28±0.57	3	0.71±0.00	1
17R-RvD1	375	215	d_5-17R-RvD1	1.15±0.76	2	1.24±0.00	1	-	-	-	-
17R-RvD3	375	147	d_5-RvD3	0.15±0.05	3	0.17±0.00	1	0.43±0.00	1	0.04±0.00	1
PD1	359	153	d_5-MaR1	0.42±0.13	7	1.09±0.00	1	0.60±0.00	1	0.87±0.17	2
17R-PD1	359	153	d_5-MaR1	0.05±0.00	1	-	-	-	-	-	-
10S,17S-diHDHA	359	153	d_5-MaR1	0.65±0.00	1	-	-	-	-	0.44±0.00	1
PCTR1	650	231	d_5-LTC$_4$	-	-	1.13±0.00	1	-	-	4.21±0.00	1
PCTR2	521	231	d_5-LTD$_4$	1.52±0.06	2	3.96±0.00	1	-	-	1.76±0.17	2
PCTR3	464	231	d_5-LTE$_4$	107.2±52.44	8	199.35±0.00	1	-	-	28.4±0.00	1
MaR1	359	221	d_5-MaR1	4.96±0.00	1	-	-	-	-	10.78±0.00	1
MaR2	359	221	d_5-MaR2	-	-	-	-	-	-	-	-
7S,14S-diHDHA	359	221	d_5-MaR1	5.89±1.92	9	1.15±0.00	1	2.34±0.00	1	6.25±3.14	3
4S,14S-diHDHA	359	101	d_5-MaR2	0.60±0.00	1	0.43±0.00	1	1.19±0.63	2	-	-
MCTR1	650	191	d_5-LTC$_4$	-	-	-	-	-	-	2.34±0.00	1
MCTR2	521	191	d_5-LTD$_4$	1.52±0.93	3	3.08±0.00	1	-	-	2.08±0.00	1
MCTR3	464	191	d_5-LTE$_4$	47.5±25.2	10	95.17±0.00	1	4.15±1.35	2	22.26±0.00	1
**n-3 DPA Bioactive Metabolome**											
RvT1	377	193	d_5-RvD2	2.79±0.48	14	1.95±1.12	3	1.80±0.52	2	1.08±0.00	1
RvT2	377	197	d_5-17R-RvD1	0.78±0.00	1	0.81±0.00	1	-	-	0.54±0.00	1
RvT3	377	173	d_5-17R-RvD1	0.25±0.00	2	0.4±0.00	1	0.38±0.00	1	-	-
RvT4	361	211	d_5-MaR1	0.94±0.20	5	-	-	-	-	-	-
RvD1$_{n-3DPA}$	377	215	d_5-17R-RvD1	0.99±0.48	2	-	-	-	-	-	-
RvD2$_{n-3DPA}$	377	233	d_5-RvD2	13.04±5.37	4	-	-	-	-	-	-
RvD5$_{n-3DPA}$	361	199	d_5-MaR1	0.77±0.26	6	3.09±0.00	1	6.04±0.00	1	1.62±0.60	4
PD1$_{n-3\ DPA}$	361	183	d_5-MaR1	0.18±0.08	4	0.08±0.00	1	0.38±0.00	1	0.40±0.17	3
PD2$_{n-3\ DPA}$	361	233	d_5-MaR2	-	-	-	-	-	-	-	-
10S,17S-diHDPA	361	183	d_5-MaR1	0.16±0.08	3	-	-	-	-	0.84±0.00	1
MaR1$_{n-3\ DPA}$	361	223	d_5-MaR1	2.16±0.00	1	-	-	2.25±0.00	1	0.28±0.00	1
MaR2$_{n-3\ DPA}$	361	223	d_5-MaR2	0.72±0.24	6	-	-	0.41±0.04	2	1.11±0.13	3
7S,14S-diHDPA	361	223	d_5-MaR1	-	-	-	-	-	-	2.31±0.00	1
**EPA Bioactive Metabolome**											
RvE1	349	161	d_4-RvE1	-	-	0.88±0.00	1	-	-	-	-
RvE2	333	159	d_4-LTB$_4$	4.76±0.00	1	-	-	-	-	-	-
RvE3	333	275	d_4-LTB$_4$	8.82±3.51	4	9.93±0.00	1	11.91±0.00	1	-	-
RvE4	333	115	d_4-LTB$_4$	7.74±2.21	19	8.13±6.98	3	1.94±0.87	5	6.80±4.64	4
**AA Bioactive Metabolome**											
LXA$_4$	351	115	d_5-LXA$_4$	0.90±0.24	6	-	-	1.72±0.70	3	0.46±0.06	3
LXB$_4$	351	221	d_5-LXA$_4$	-	-	-	-	-	-	1.72±0.72	3
5S,15S-diHETE	335	235	d_4-LTB$_4$	7.28±2.44	12	10.26±5.88	2	0.78±0.31	2	10.55±5.22	4
15-epi-LXA$_4$	351	115	d_5-LXA$_4$	1.83±0.46	6	-	-	1.27±0.44	3	0.31±0.00	1
15-epi-LXB$_4$	351	221	d_5-LXA$_4$	45.46±30.71	4	1.76±0.39	3	3.19±0.00	1	2.77±0.69	2
LTB$_4$	335	195	d_4-LTB$_4$	1.12±0.26	16	2.82±1.44	3	2.95±1.84	6	2.68±1.07	6
5S,12S-diHETE	335	195	d_4-LTB$_4$	0.86±0.16	13	0.77±0.32	3	1.18±0.46	5	1.36±0.09	3
6-trans-LTB$_4$	335	195	d_4-LTB$_4$	0.52±0.13	11	0.32±0.25	2	0.52±0.24	3	-	-
6-trans-12-epi LTB$_4$	335	195	d_4-LTB$_4$	0.67±0.16	9	1.37±0.49	2	0.32±0.09	3	3.93±1.03	3
LTC$_4$	626	189	d_5-LTC$_4$	6.65±0.00	1	-	-	-	-	-	-
LTD$_4$	497	189	d_5-LTD$_4$	0.52±0.06	3	1.22±0.00	1	1.51±0.00	1	0.54±0.00	1
LTE$_4$	440	189	d_5-LTE$_4$	1.50±0.24	13	1.40±0.12	3	2.85±0.98	6	2.64±0.45	7
PGD$_2$	351	189	d_4-PGE$_2$	22.05±10.88	18	9.30±6.80	2	11.51±4.03	4	9.10±4.89	7
PGE$_2$	351	189	d_4-PGE$_2$	8.16±5.82	13	0.40±0.26	3	1.68±0.93	3	0.79±0.51	6
PGF$_{2\alpha}$	353	193	d_4-PGE$_2$	12.45±8.66	11	2.29±1.49	2	0.66±0.14	3	3.44±2.49	6
TXB$_2$	369	169	d_4-PGE$_2$	297.4±171.6	10	55.0±26.4	3	238.9±204.3	2	26.23±21.13	5

Results are reported as pg mediator/mL of plasma for all samples in which that mediator was identified. n=21 for Improving/Stable WHO 3-4 patients, n=4 for Deteriorating WHO 3-4 patients, n=6 for Improving/Stable WHO 5 patients, n=7 for Deteriorating WHO 5 patients.

Supplemental Table IX: Lower limits of the lipid mediator profiling assay

DHA Bioactive Metabolome	Q1	Q3	pg
RvD1	375	215	0.20
RvD2	375	141	0.10
RvD3	375	147	0.05
RvD4	375	101	0.50
RvD5	359	199	0.10
RvD6	359	101	n.d.
17R-RvD1 *	375	215	0.20
17R-RvD3 *	375	147	0.05
PD1	359	153	0.10
17R-PD1 *	359	153	0.10
10S,17S-diHDHA *	359	153	0.10
PCTR1	650	231	0.50
PCTR2	521	231	0.10
PCTR3	464	231	0.50
MaR1	359	221	0.50
MaR2	359	221	0.20
7S,14S-diHDHA *	359	221	0.50
4S,14S-diHDHA *	359	101	n.d.
MCTR1	650	191	1.00
MCTR2	521	191	0.10
MCTR3	464	191	0.05
**n-3 DPA Bioactive Metabolome**	**Q1**	**Q3**	**pg**
RvT1	377	193	0.20
RvT2	377	197	n.d.
RvT3	377	173	n.d.
RvT4	361	211	0.20
$RvD1_{n\text{-}3DPA}$	377	215	0.20
$RvD2_{n\text{-}3DPA}$	377	233	5.00
$RvD5_{n\text{-}3DPA}$	361	199	0.10
$PD1_{n\text{-}3\ DPA}$	361	183	n.d.
$PD2_{n\text{-}3\ DPA}$	361	233	n.d.
10S,17S-diHDPA	361	183	n.d.
$MaR1_{n\text{-}3\ DPA}$	361	223	n.d.
$MaR2_{n\text{-}3\ DPA}$	361	223	n.d.
7S,14S-diHDPA	361	223	n.d.
**EPA Bioactive Metabolome**	**Q1**	**Q3**	**pg**
RvE1	349	161	0.20
RvE2	333	159	0.10
RvE3	333	275	5.00
RvE4	333	115	n.d.
**AA Bioactive Metabolome**	**Q1**	**Q3**	**pg**
LXA_4	351	115	0.10
LXB_4	351	221	0.20
5S,15S-diHETE	335	235	0.10
15-epi-LXA_4 *	351	115	0.10
15-epi-LXB_4 *	351	221	0.20
LTB_4 *	335	195	0.20
5S,12S-diHETE	335	195	0.20
6-trans-LTB_4 *	335	195	0.20
6-trans-12-epi LTB_4 *	335	195	0.20
LTC_4	626	189	0.50
LTD_4	497	189	0.20
LTE_4	440	189	0.10
PGD_2	351	189	0.05
PGE_2	351	189	0.05
$PGF_{2\alpha}$	353	193	0.20
TXB_2	369	169	0.20

Internal Standards	Q1	Q3	pg
d_4-LTB_4	339	197	0.20
d_5-MaR1	364	177	0.50
d_5-MaR2	364	177	0.50
d_4-PGE_2	355	193	0.05
d_5-LXA_4	356	115	0.10
d_5-RvD3	380	147	1.00
d_5-RvD2	380	141	0.50
d_4-RvE1	353	197	0.20
d_5-17R-RvD1	380	141	0.25
d_5-LTC_4	631	189	0.50
d_5-LTD_4	502	189	0.05
d_5-LTE_4	445	189	0.10

Limits were determined in charcoal stripped plasma. * Denotes mediators where isomers (chiral or double bond) were used to establish the limits using the same transition as used for quantitation. This is the list of standards with their respective transition that were used as surrogates: RvD1 (375/215) for 17R-RvD1; RvD3 (375/147) for 17R-RvD3; PD1 (359/153) for 17R-PD1, 10S,17S-diHDHA and 10S,17S-diHDPA; MaR1 (359/221) for 7S,14S-diHDHA; LXA4 (351/115) for 15-epi-LXA_4; LXB_4 (351/221) for 15-epi-LXB_4; d_4-LTB_4 (339/197) for LTB_4, 5S,12S-diHETE, 6-trans-LTB_4, and 6-trans-12-epi-LTB_4. n.d. = not determined. These are mediators where synthetic standards were not available to establish the limits.

Supplemental Figure I

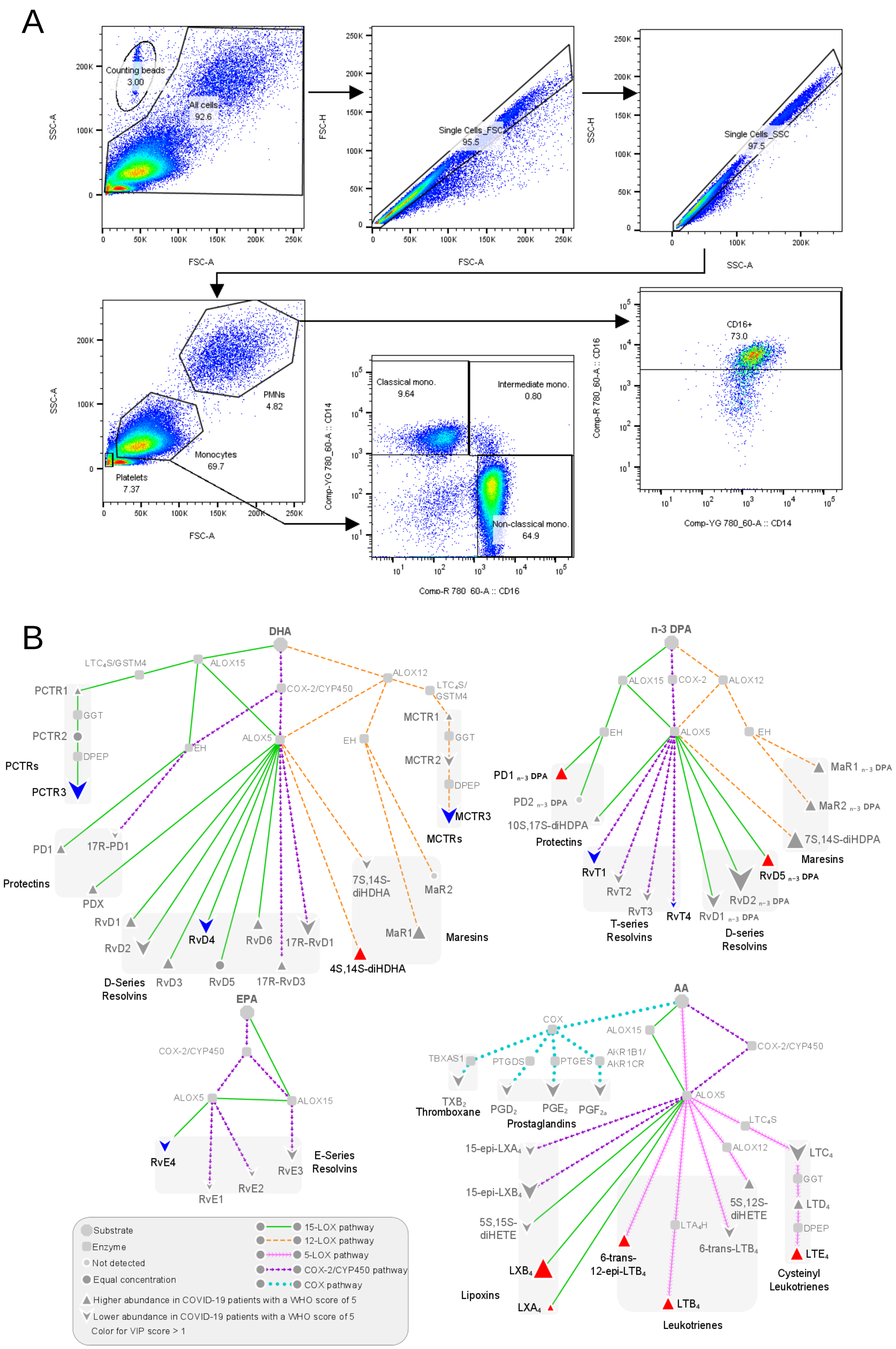

Supplemental Figure I: (A) Gating strategy used to identify different phagocyte subsets in peripheral blood. Cell populations were identified according to their characteristic FSC/SSC profile, doublets were excluded, and cell surface marker expression was evaluated by flow cytometry, with neutrophils defined as CD16^{+}, classical monocytes as CD14^{+}CD16^{+}, intermediate monocytes as CD14^{++}CD16^{++}, non-classical monocytes as CD14^{+}CD16^{++}, and platelets as CD41a^{+}. At least 100.000 events were recorded in the first gate ("All cells") for all samples, resulting in ~5.000-10.000 events in the final neutrophil and monocyte subset gates. (B) Analysis of lipid mediators in plasma from COVID-19 patients at WHO scale 3-4 and WHO scale 5, highlighting (red or blue) mediators with VIP score >1 in PLS-DA and their biosynthetic pathways. Results are from n=25 WHO 3-4 patients and n=14 WHO 5 patients.

Supplemental Figure II

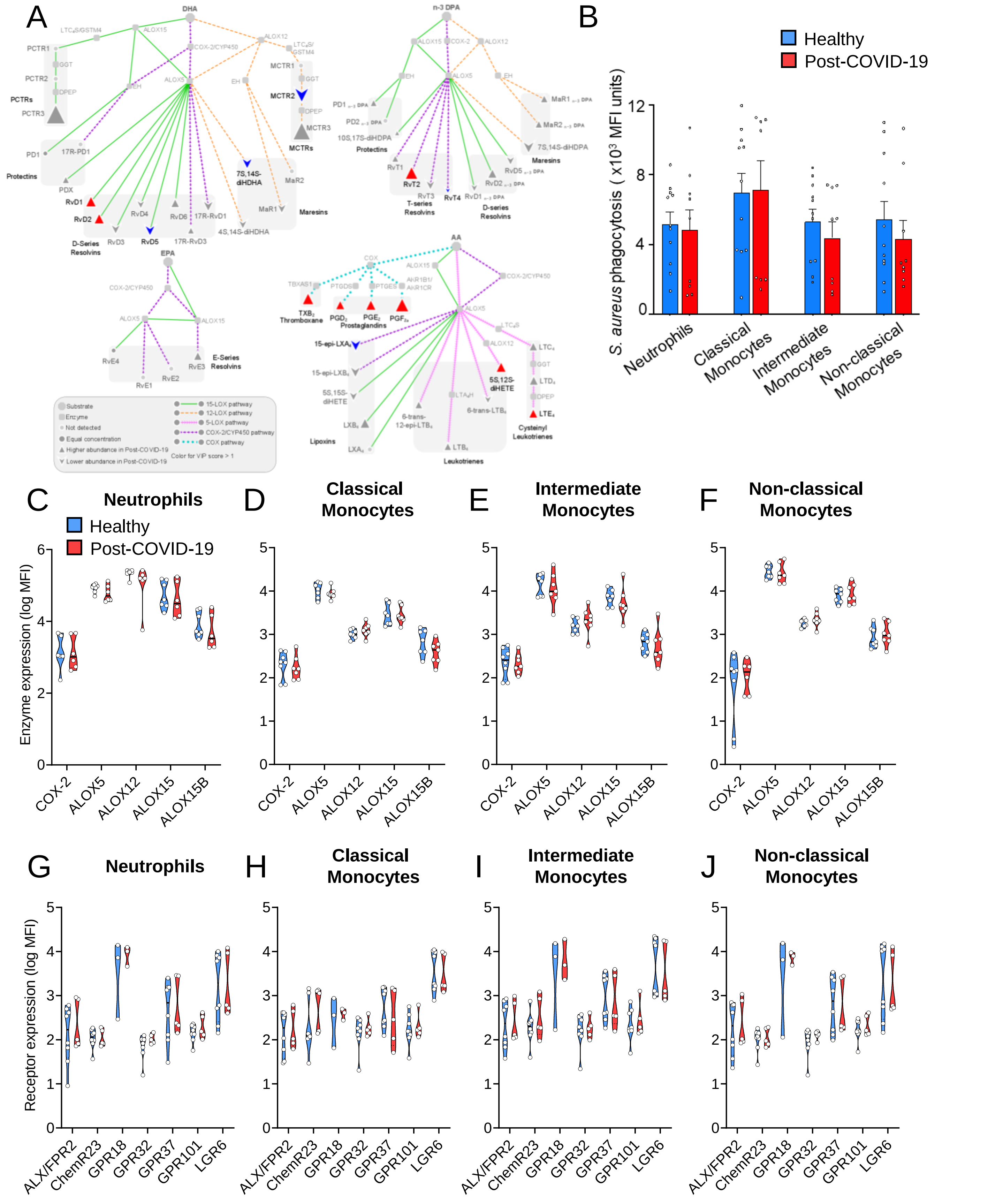

Supplemental Figure II: Altered phagocyte activation status persists after the resolution of COVID-19 symptoms. (A) Analysis highlighting the mediators that were found to be differentially regulated in PLS-DA analysis and their biosynthetic pathways. Results are from n=12 Healthy volunteers and n=9 post-COVID-19 volunteers. (B) Peripheral blood was incubated with fluorescently labelled *S. aureus* for 60 minutes then with lineage markers for neutrophils and monocyte subsets and bacterial uptake was evaluated using flow cytometry. n=11 healthy volunteers and n=9 post-COVID-19 volunteers. Statistical differences were evaluated using Mann-Whitney Test. (C-J) Peripheral blood was collected from volunteers after the resolution of COVID-19 clinical symptoms (post-COVID-19) or healthy volunteers and incubated with fluorescently conjugated antibodies against lineage markers for neutrophils and monocyte subsets as well as fluorescently conjugated antibodies against (C-F) lipid mediator biosynthetic enzymes or (G-J) SPM receptors and the expression was evaluated using flow cytometry. For (C), n=6 Healthy volunteers and n=6 post-COVID-19 volunteers. For (D-F), n=7 Healthy volunteers and n=6 post-COVID-19 volunteers. For (G-J), n=7 Healthy volunteers and n=5 post-COVID-19 volunteers (except for GPR18, where n=3 for both Healthy and post-COVID-19 volunteers. Statistical differences were evaluated using Mann-Whitney Test.

Supplemental Figure III

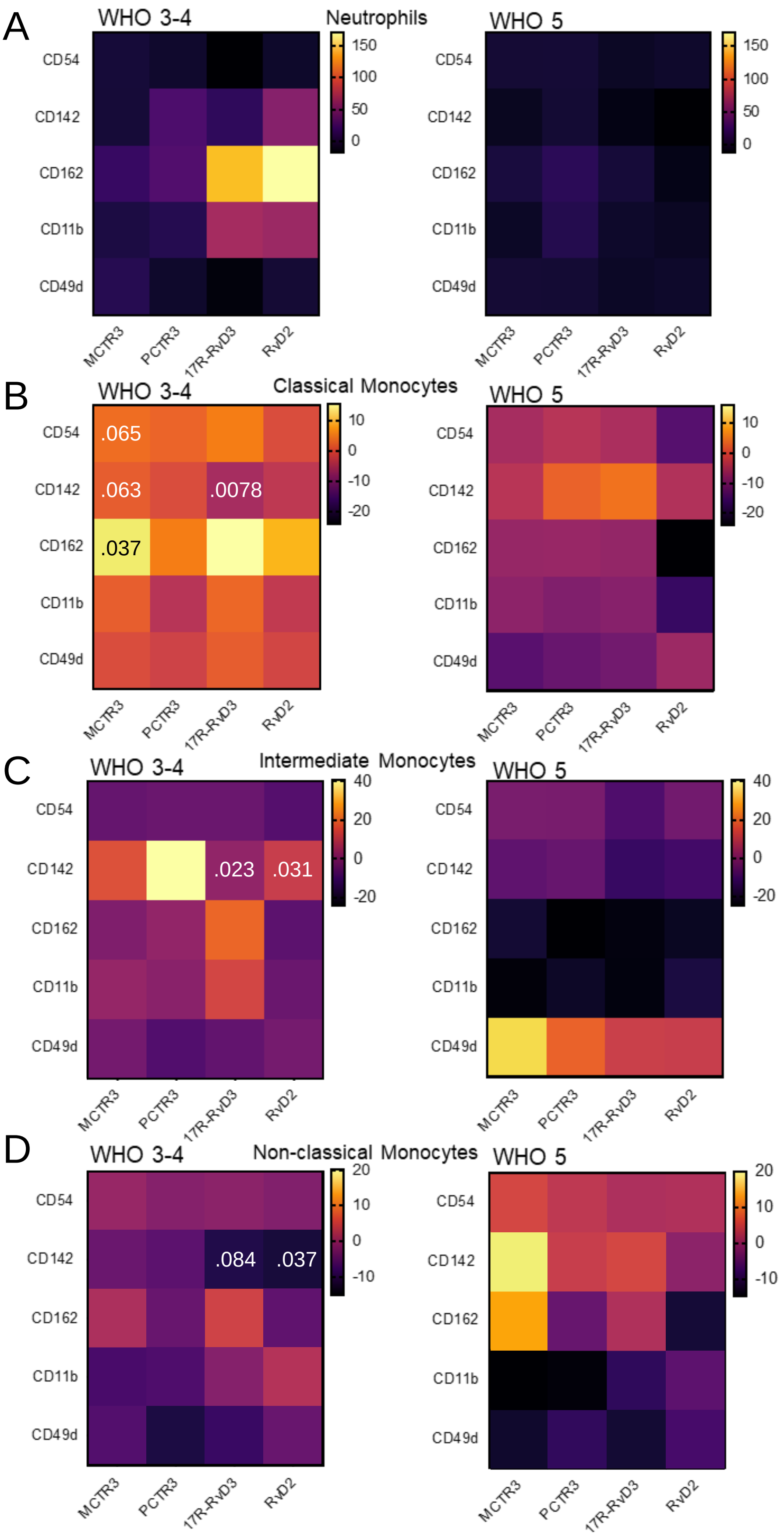

Supplemental Figure III: Effect of SPM on expression of activation markers in peripheral blood phagocytes from COVID-19 patients with mild/moderate and severe disease. Neutrophils and monocytes were isolated from peripheral blood of COVID-19 patients with either mild/moderate (WHO scale of 3 or 4; WHO3-4) or those with severe disease (WHO scale of 5; WHO5) using density centrifugation. Cells were then incubated with 1nM of the indicated SPM or vehicle for 60 minutes and the expression of adhesion molecules in (A) neutrophils (B) Classical monocytes (C) Intermediate monocytes (D) Non-classical monocytes was assessed using flow cytometry. Results are reported as percentage change from expression levels in cells incubated with vehicle only (0.01% EtOH) and are representative of n=4 for neutrophils from patients at WHO scale 3-4 and n=6 patients at WHO scale 5. For monocyte subsets n=11 for patients at WHO scale 3-4 and n=6 patients at WHO scale 5. Statistical differences were established using Wilcoxon Signed Rank test and raw p-values are displayed.

Supplemental Figure IV

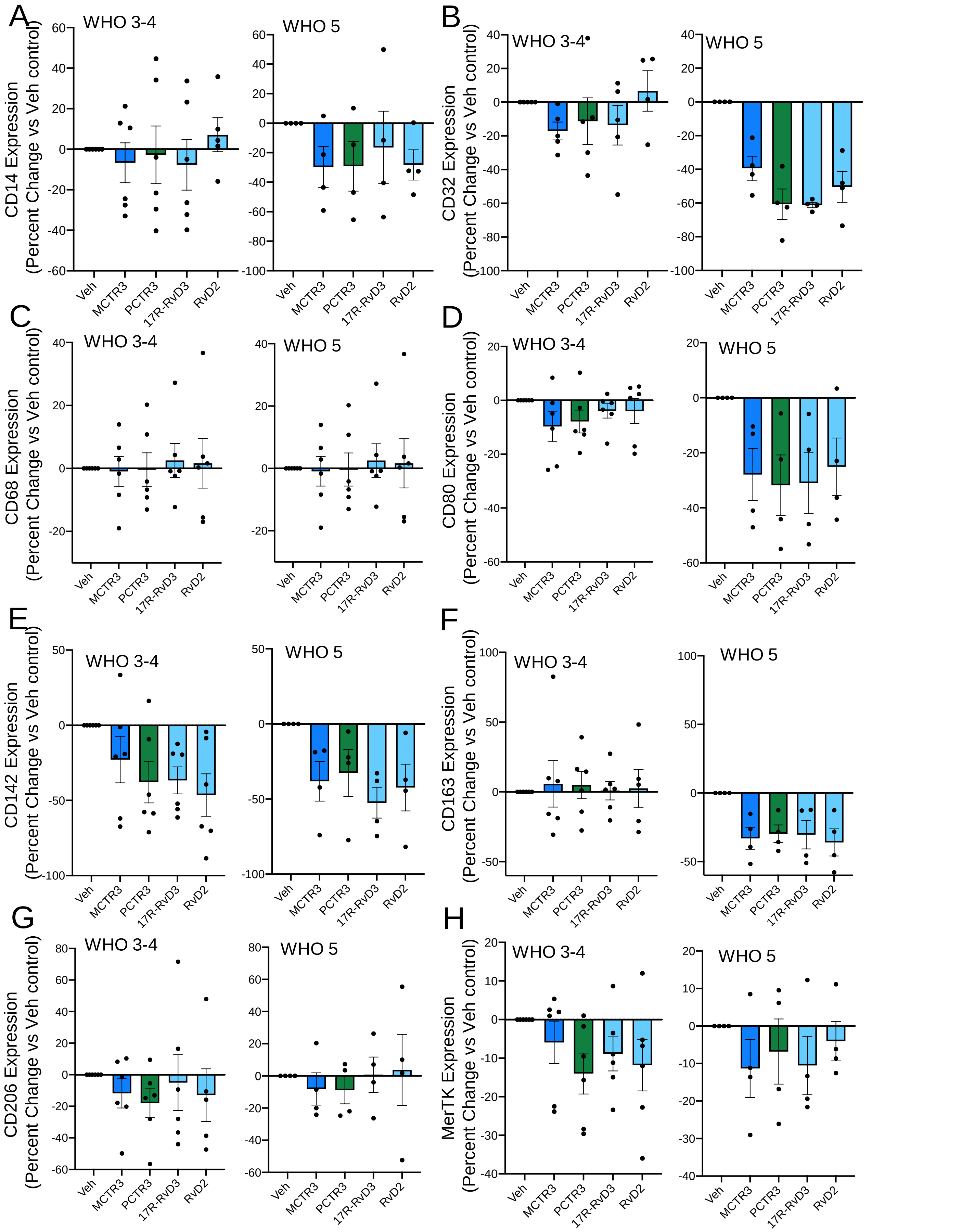

Supplemental Figure IV: Regulation of phenotypic markers in monocyte-derived macrophages from COVID-19 patients with mild/moderate and severe disease. (A-H) Monocytes were isolated from peripheral blood of patients with COVID-19 and differentiated to monocyte-derived macrophages using GM-CSF in the presence of vehicle or 10nM of the indicated SPM. On day 7, cells were lifted and the expression of the indicated phenotypic markers was assessed using flow cytometry. Results are reported as percentage change in expression from levels in cells incubated with vehicle only and are representative of n=7 for patients at WHO scale 3-4 and n=6 at WHO scale 5 score.

Supplemental Figure V

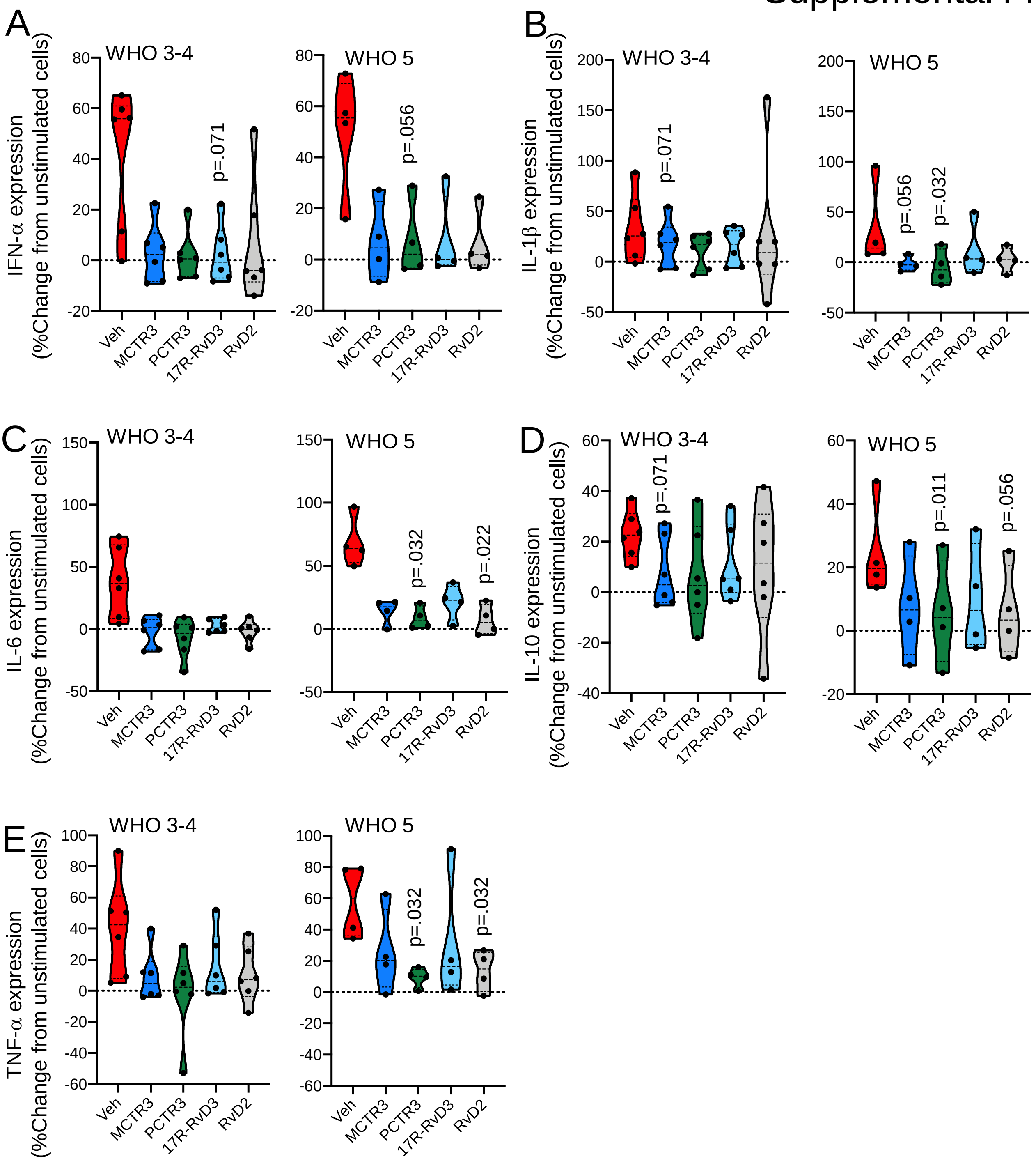

Supplemental Figure V: SPM downregulate pro-inflammatory cytokine production in monocyte-derived macrophages from patients with both mild/moderate and severe disease. Monocytes were isolated from peripheral blood of patients with COVID-19 and differentiated to monocyte-derived macrophages with GM-CSF in the presence of vehicle or 10nM of the indicated SPM. On day 7, cells were incubated with recombinant human S100A8/A9 dimer (1 µg/ml, 24 hours) and Brefeldin A (2 µg/ml, for final 18 hours) and the expression of the indicated cytokines was assessed using flow cytometry. Results are reported as percentage change in expression from levels in cells incubated with vehicle only and are representative of n=7 for patients at WHO scale 3-4 and n=6 at WHO scale 5. Statistical differences were established using Kruskal-Wallis test with Dunn's post-hoc correction.

Supplemental Figure VI

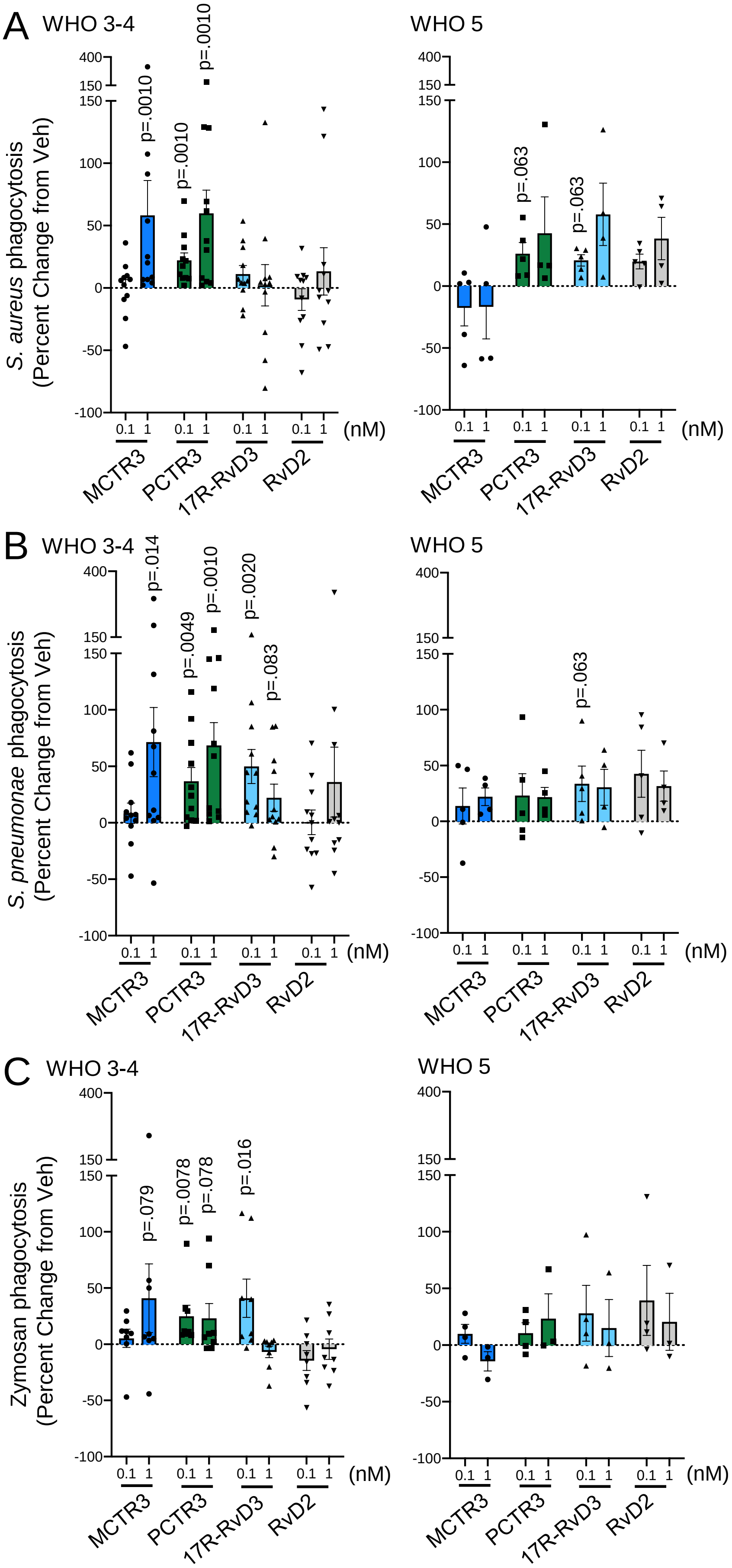

Supplemental Figure VI: Upregulation in bacterial and fungal phagocytosis by SPM in patient with both mild/moderate and severe disease. Monocytes from COVID-19 patients were differentiated to monocyte-derived macrophages with GM-CSF and incubated with the indicated SPM (0.1 or 1 nM) or vehicle for 15 min followed by fluorescently labelled (A) *S. aureus*, (B) *S. pneumoniae*, or (C) zymosan. Results are reported as percentage change in phagocytosis from levels in cells incubated with vehicle only and are representative of (A-B) n=11 for patients at WHO scale 3-4 and n=5 for patients at WHO scale 5 incubated with *S. aureus* or *S. pneumoniae*. (C) n=9 for patients at WHO scale 3-4 and n=5 patients at WHO scale 5 incubated with zymosan. Statistical differences were established using Wilcoxon Signed Rank test and raw p-values are displayed.
